# Drosophila Spaghetti and Doubletime Link the Circadian Clock and Light to Caspases, Apoptosis and Tauopathy

**DOI:** 10.1371/journal.pgen.1005171

**Published:** 2015-05-07

**Authors:** John C. Means, Anandakrishnan Venkatesan, Bryan Gerdes, Jin-Yuan Fan, Edward S. Bjes, Jeffrey L. Price

**Affiliations:** 1 Division of Molecular Biology and Biochemistry, School of Biological Sciences, University of Missouri-Kansas City, Kansas City, Missouri, United States of America; 2 Department of Neurology and Cognitive Neuroscience, School of Medicine, University of Missouri-Kansas City, Kansas City, Missouri, United States of America; Washington University Medical School, UNITED STATES

## Abstract

While circadian dysfunction and neurodegeneration are correlated, the mechanism for this is not understood. It is not known if age-dependent circadian dysfunction leads to neurodegeneration or vice-versa, and the proteins that mediate the effect remain unidentified. Here, we show that the knock-down of a regulator (*spag*) of the circadian kinase Dbt in circadian cells lowers Dbt levels abnormally, lengthens circadian rhythms and causes expression of activated initiator caspase (Dronc) in the optic lobes during the middle of the day or after light pulses at night. Likewise, reduced Dbt activity lengthens circadian period and causes expression of activated Dronc, and a loss-of-function mutation in *Clk* also leads to expression of activated Dronc in a light-dependent manner. Genetic epistasis experiments place Dbt downstream of Spag in the pathway, and Spag-dependent reductions of Dbt are shown to require the proteasome. Importantly, activated Dronc expression due to reduced Spag or Dbt activity occurs in cells that do not express the *spag* RNAi or dominant negative Dbt and requires PDF neuropeptide signaling from the same neurons that support behavioral rhythms. Furthermore, reduction of Dbt or Spag activity leads to Dronc-dependent Drosophila Tau cleavage and enhanced neurodegeneration produced by human Tau in a fly eye model for tauopathy. Aging flies with lowered Dbt or Spag function show markers of cell death as well as behavioral deficits and shortened lifespans, and even old wild type flies exhibit Dbt modification and activated caspase at particular times of day. These results suggest that Dbt suppresses expression of activated Dronc to prevent Tau cleavage, and that the circadian clock defects confer sensitivity to expression of activated Dronc in response to prolonged light. They establish a link between the circadian clock factors, light, cell death pathways and Tau toxicity, potentially via dysregulation of circadian neuronal remodeling in the optic lobes.

## Author Summary

Alzheimer’s disease is the most common cause of dementia in the aging population. It is a progressive neurodegenerative disorder that attacks the brain neurons, resulting in loss of memory, thinking and behavioral changes. One pathological hallmark is aggregation of the microtubule-associated protein Tau. A growing body of evidence highlights the importance of caspase-dependent Tau truncation in initiation and potentiation of Tau aggregation. Here we use the fruit fly Drosophila to examine the links between circadian rhythms, aging, apoptosis and Alzheimer’s Disease. We identified a regulator (*spag)* of the circadian kinase Dbt that functions to stabilize Dbt during the middle of the day. In addition, the caspase Dronc is regulated by Dbt and Spag and, when activated by reduction of either, targets Tau for cleavage, leading to behavioral deficits and shortened lifespans. The expression of activated caspase occurs in several parts of the brain in a manner requiring signaling from a neuropeptide produced by circadian cells. Wild type flies with no genetic modifications eventually exhibit modified Dbt and expression of activated caspase at specific times of day, further demonstrating the links between the circadian clock, light and apoptosis.

## Introduction

Alzheimer’s disease (AD) is a neurodegenerative disorder that involves neuronal cell loss, extracellular amyloid plaques, and intracellular neurofibrillary tangles. During AD and other neurodegenerative diseases, neurons induce a series of proteases, including caspases, and a number of key proteins are cleaved by caspases including APP, Presenilin (PS1, PS2), Tau and Huntingtin [1–5]. This has led to the suggestion that the extensive neuronal loss observed in AD may result from the activation of apoptotic related pathways [6]. Caspases can be activated within a cell without immediately causing classical apoptosis, and there is evidence for prolonged caspase activation without neuronal death [7]. For instance, in AD chronic caspase activation may lead to cleavage of Tau and other essential cellular proteins and contribute to neuronal pathology prior to cell death [3]. Caspase-cleaved Tau is present in AD, but not control brain [8,9]. Additionally, caspase-cleaved Tau is more fibrillogenic in vitro than full-length Tau [9].

*Drosophila melanogaster* has been used as a model system for the analysis of AD [10]. The Drosophila Tau protein is similar to the mammalian one and accumulates in axonal processes [11]. When it is overexpressed in neurons or eyes, fly Tau induces apoptotic neuronal cell death [12]. In addition, synaptic dysfunctions induced by human or fly Tau have been produced and analyzed in Drosophila larval motor neurons and neuromuscular junctions. Expression of Tau within motor neurons generates altered morphology in the presynaptic terminals and defective synaptic transmission and microtubule-based axonal transport [13,14]. The fly mushroom body is the key locus for olfactory learning and memory in Drosophila, where human or fly Tau expression causes an impairment of associative learning and memory followed by neurodegeneration [15]. Finally, when expressed in the fly eye or brain, human Tau produces aspects of human tauopathies, including neurodegeneration [16,17].

Like many organisms, Drosophila operates on a 24-hour cycle that is maintained by environmental input to an internal body clock [18]. The clock depends on oscillations in the activation of specific genes at certain times of the day. The key feature of these oscillations is a negative feedback loop, in which transcriptional regulators like Period (Per) repress transcription of their mRNAs. The *Drosophila* Doubletime (Dbt) protein is homologous to mammalian Casein Kinase 1 and phosphorylates Per monomers, resulting in Per degradation [19,20]. In addition to its role in regulating circadian rhythms, Dbt has also been shown to be involved in regulating cell death pathways. For example, overexpression of Dbt in the fly eye has been shown to rescue the eye morphology defect caused by expression of proapoptotic proteins such as Reaper and Hid [21].

Circadian rhythm disturbances affect as many as 25% of AD patients during some stage of their disease [22]. As a consequence, sleep, the biological clock, and core body temperature are affected. Some common symptoms of AD that are related to disturbances in the circadian clock are insomnia, nocturnal behavioral changes, and excessive daytime sleepiness. A postmortem study found significant differences in the expression pattern of circadian genes between Alzheimer patients and controls [23]. In addition, the 3xTg (triple transgenic) mouse models of AD, which exhibit Tau neuropathology, showed deteriorated circadian organization of locomotor behavior [24]. However, it is not known if circadian components are directly linked to disease onset or if circadian dysfunction is just a consequence of AD.

Our search for proteins that interact with the Drosophila circadian kinase Dbt has led to results demonstrating that Dbt and one of these interactors connect the circadian, cell death and neurodegenerative pathways. Initially, a screen was performed for effectors of Dbt’s circadian function; we screened a list of candidate genes (mostly phosphatase catalytic subunits) with dsRNAi lines crossed to the timGAL4 driver for those that would alter circadian periods and lead to changes in Dbt electrophoretic mobility, potentially indicative of those that would lead to autophosphorylation of Dbt. This screen identified *spaghetti* (CG13570, or *spag*). *spag* encodes a tetratricopeptide repeat (TPR)-containing protein initially identified in a screen for modifiers of protein aggregation in Huntington disease and was reported to interact both with Huntingtin protein and the chaperone protein HSP90 [25]. Here, we show that *spag* knock-down or reduced Dbt activity in circadian cells leads to longer circadian periods and expression of activated initiator caspase only during the middle of the day. Links to the circadian clock are shown by the effects of light, circadian mutants and circadian cells, while links to AD are shown by the resulting cleavage of Tau, enhancement of neurodegeneration, reduced healthspan and effects of aging.

## Results

### *Spaghetti* knockdown leads to circadian rhythm disruption, reductions in Dbt, and Dbt-dependent caspase activation after extended light treatment

We targeted the clock cells in flies with *spag* RNAi knock-down (*tim*GAL4>UAS-*dcr*; UAS-*spag* RNAi) and observed altered locomotor activity rhythms. *spag* knockdown flies exhibited long periods (25.5–26.5 h) or arrhythmic locomotor activity (S1 Fig and Table 1), depending on the specific RNAi transgene, the inclusion of *dcr* in the genotype and the duration of transgene expression after eclosion. Null mutations of *spag* and ubiquitous knock-down of *spag* were lethal, so all of our analysis has employed knock-downs of *spag* in circadian cells with the *tim*GAL4 or *pdf*GAL4 drivers.

**Table 1:** Locomotor activity rhythms of flies with *spag* RNAi knockdown.

Week	Genotype	Avg Period (h) ± SEM	% Rhythmicity (n)
**2**	**UAS-*dcr2*; *tim*GAL4>/CyO**	24.5 ± 0.2	61 (28)
	**Wild type (Canton S)**	23.6 ± 0.06	87 (15)
	**UAS-*dcr2*; *tim*GAL4>/23896**	26.3 ± 0.1**	67 (43)
	**UAS-*dcr2*; *tim*GAL4>/31253**		0 (5)^1^
	**UAS-*dcr2*; *tim*GAL4>/103353KK**		0 (0)^2^
	***tim*GAL4>/31253**	24.2 ± 0.4	20 (15)
	***tim*GAL4>/103353KK**	25.2 ± 0.3*	45 (20)
**1**	**Wild type (Canton S)**	23.6± 0.2	97 (32)
	**103353KK**	23.8 ± 0.3	91 (11)
	**31253**	23.6 ± 0.1	80 (10)
	**UAS-*dcr2*; *tim*GAL4>/31253**	24.8 ± 0.5	56 (9)
	**UAS-*dcr2*; *tim*GAL4>/103353KK**	25.0 ± 0.5	20 (10)
	***tim*GAL4>/31253**	23.4 ± 0.08	40 (15)
	***tim*GAL4>/103353KK**	24.3 ± 0.2	47 (15)

^**1**^11 of 16 flies died during the locomotor assay.

^2^16 of 16 flies died during the locomotor assay.

The *tim*GAL4 or UAS-*dcr*; *tim*GAL4 lines were crossed to the indicated responder line (stock number from VDRC or Bloomington Stock Center indicated), entrained to LD 12hr:12hr, and male progeny hemizyous for UAS-*dcr* (where indicated) and heterozygous for *tim*GAL4 and the responder were collected after 18 days. These were immediately placed into activity assays that were monitored for one week in DD (1 week assays) or aged for 1 week and then monitored for another week in DD (2 week assays). The periods were determined by chi-square periodogram analysis, and rhythmic flies produced a single strong peak above the line of statistical significance (p<0.01) and clear rhythmicity by visual inspection of the actograms. The average periods of rhythmic flies are tabulated here, along with the % rhythmicity (n is the number of flies tested). The effect of group on period was statistically significant by one-way ANOVA (F(10, 129) = 34.342, p<0.001). The difference from all groups (**) or from the relevant controls (*, Canton S and responders only) was significant by post-hoc Tukey (p<0.05).

**Fig 1:**
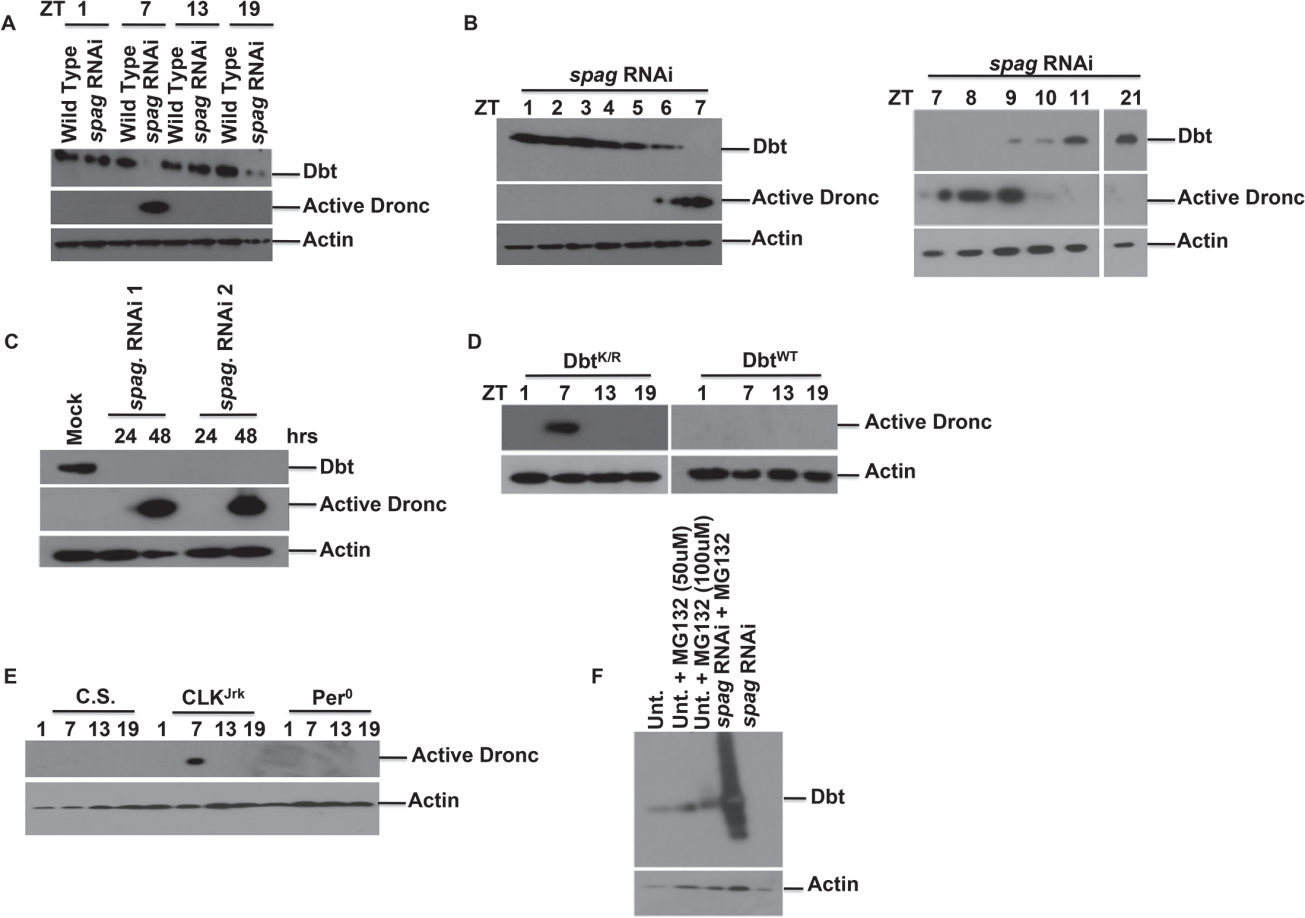
Knock-down of *spaghetti* reduces Dbt and causes accumulation of activated initiator caspase at ZT7. (A) Wild type Canton S or *tim*GAL4>UAS-*spag* RNAi fly heads were harvested at various times as indicated (ZT: hours after lights on in a 12hr:12hr light:dark cycle) and immunoblotted for Dbt, active Dronc and actin as a loading control. (B) *tim*GAL4>*spag* RNAi fly heads were harvested at the indicated times in LD (ZT) and immunoblotted for Dbt or active Dronc. (C) S2 cells were harvested 24 and 48 hours after *spag* dsRNA addition (two different nonoverlapping dsRNAis were used) and immunoblotted for Dbt, active Dronc and actin. (D) *tim*GAL4>UAS-*dbt* ^K/R^ and-*dbt* ^WT^ fly heads were harvested at the indicated times and immunoblotted for active Dronc and actin. (E) *Clk* ^*Jrk*^ flies express activated Dronc at ZT 7. Fly heads from various clock mutants or wild type flies were collected at the indicated times and analyzed for activated caspase expression. (F) Reduced Dbt levels caused by RNAi knock-down of *spag* are caused by the proteasome. S2 cells were untreated or treated with *spag* dsRNA +/- MG132 (a proteasome inhibitor) and immunoblotted for Dbt and actin.

In addition to locomotor defects, knockdown of *spag* led to a decrease in Dbt protein levels and an increase in the levels of activated initiator caspase Dronc, detected with an antibody that only detects the cleaved (and thereby activated) form of Dronc [26] (Figs 1A and S2A; See Materials and Methods). Interestingly, the decrease in Dbt and the accumulation of activated Dronc occurred mostly during the daytime close to ZT7 (Lights are on from ZT0-12 and off from ZT12-24). To determine the time course more precisely, head extracts were collected at one hour intervals from ZT1-7, and Dbt disappearance and activated caspase were detected by ZT6 (Fig 1B). In addition, the levels of activated Dronc were reduced after ZT9 and gone by ZT11 (Fig 1B). These effects of *spag* knock-down were further confirmed in Drosophila S2 cells, which lack a circadian rhythm, and knockdown of *spag* with two different non-overlapping dsRNAi’s led to a decrease in Dbt levels, followed by accumulation of activated Dronc (Figs 1C and S3A). The loss of Dbt from Drosophila S2 cells or fly heads was preceded by a post-translational modification of Dbt that produced a mobility shift with SDS-PAGE (S2B–S2E Fig), and in fly heads the loss was not always complete but was enhanced at higher temperatures, longer times post-eclosion and by light rather than darkness (S2C, S2D, and S2E Fig). Light is a strong trigger, as Dronc was activated 7 hrs after light periods starting at ZT13 in a number of lines (including *spag* RNAi) that produced activated Dronc during the day (S2F Fig). A variable amount of Dbt reduction was also observed in the middle of the night (ZT19; Figs 1A and S3B), although activated Dronc was not detected. In summary, reduced *spag* levels in circadian cells triggers post-translational modification of Dbt, reduced Dbt levels and accumulation of activated caspase at specific times of day.

To address whether the caspase activation was a consequence of loss of Dbt function we expressed the kinase-dead (Dbt^K/R^) form of Dbt in circadian cells with a *tim*GAL4 driver. The Dbt^K/R^ protein acts as a dominant negative to antagonize endogenous Dbt [27]. Dbt^K/R^ flies also showed elevated caspase activity at ZT7, while flies expressing Dbt^WT^ did not (Fig 1D). The results suggest that the kinase activity of Dbt negatively regulates expression of activated Dronc, and that reductions in this activity can cause expression of activated Dronc.

To determine if other clock mutants showed activated caspase expression we collected heads of *per* ^*0*^ and *Clk* ^*Jrk*^ mutant flies and analyzed for activated caspase expression. *Clk* ^*Jrk*^ flies showed activated caspase expression at ZT7 like flies expressing Dbt^K/R^, but *per* ^*0*^ did not (Fig 1E), and more extensive analysis of *Clk* ^*Jrk*^ flies showed activated caspase only at ZT7-9 in *Clk* ^*Jrk*^ flies (S4 Fig). *tim*GAL4>UAS-*dbt* ^*K/R*^ flies express high levels of PER [27], which should repress the CLK/CYC transcription factor and thereby produce a condition like that found in *Clk* ^*Jrk*^ flies, which lack CLK-dependent transcription. Therefore, activated caspase is produced in two different circadian mutants with similar effects on the circadian transcription cycle. In order to determine if the time of the circadian clock (e.g., ZT7) is needed for production of activated Dronc, we examined the timing of Dronc induction in *per* ^*S*^ (or *per* ^*L*^); *tim*GAL4>UAS-*dbt* ^*K/R*^ flies, along with its activation in *Clk* ^*Jrk*^ flies and wild type flies, and in all mutant flies activation was detected from ZT7-9 (S4 Fig), despite the fact that the *per* ^*S*^ mutation significantly shortened the period of locomotor activity for the *tim*GAL4>UAS-*dbt* ^*K/R*^ genotype (31.0 ± 0.4, n = 14 vs 33.7 ± 0.9, n = 15 for the *per* ^*S*^ and *per* ^*+*^; *dbt* ^*K/R*^ genotypes respectively; all *per* ^*L*^ *; dbt* ^*K/R*^ flies, n = 16, were arrhythmic). Taken together with the ZT7 time of Dronc activation for the largely arrhythmic *tim*GAL4>UAS-*dbt* ^*K/R*^ and *Clk* ^*Jrk*^ genotypes, the absence of Dbt reductions in DD (S2E Fig), and the production of activated Dronc in light-pulsed flies at night (S2F Fig), these results suggest that the production of activated caspase is a transient response to prolonged light exposure that also involves reductions in several circadian genes (e.g., *spag*, *dbt* and *clk*).

### Dbt is targeted for degradation via the proteasome

S2 cells were treated with *spag* dsRNA with and without the proteasome inhibitor MG132 and immunoblotted for Dbt. Endogenous Dbt levels were stabilized in the presence of the proteasome inhibitor in the presence of *spag* RNAi, which led to complete absence of Dbt in the absence of proteasome inhibitor, but higher Dbt levels in the presence of proteasome inhibitor and *spag* RNAi were obtained than with proteasome inhibitor only (Fig 1F). The higher levels of Dbt in the presence of *spag* RNAi and proteasome inhibitor than with proteasome inhibitor only, coupled with the lower levels of Dbt with *spag* RNAi only, suggest that Spag may have both proteasome-dependent positive and proteasome-independent negative effects on Dbt levels. This result also demonstrates that Dbt is degraded by the proteasome in response to *spag* knock-down. The transient accumulation of forms of Dbt with slow electrophoretic mobility (S2B–S2E Fig) suggests that phosphorylation and ubiquitination of Dbt is the initial consequence of the *spag* knock-down (See [28,29] for evidence of phosphorylation.), with proteasomal degradation of Dbt a subsequent consequence.

### Dbt^WT^ can rescue *spag* RNAi-induced caspase activation

**Fig 2:**
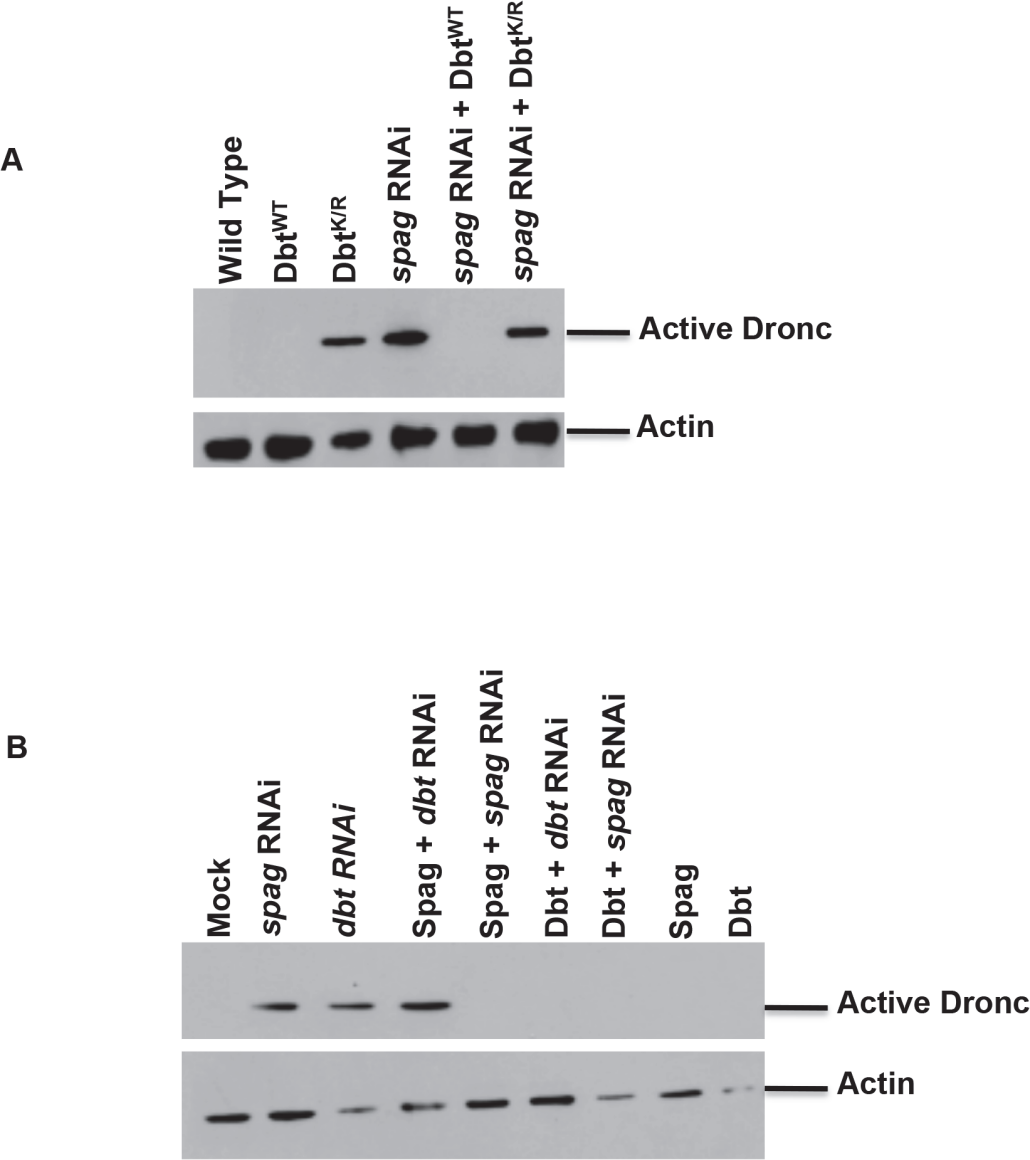
Overexpression of Dbt^WT^ can rescue the caspase activation associated with *spag* RNAi. (A) Fly heads expressing Dbt^WT^, Dbt^K/R^, *spag* RNAi (31253), *spag* RNAi+Dbt^K/R^ or *spag* RNAi+Dbt^WT^ with the *tim*GAL4 driver were collected at ZT7 and analyzed for active Dronc or actin. (B) S2 cells stably overexpressing Spag or Dbt^WT^ were treated with *spag* or *dbt* dsRNA and immunoblotted for active Dronc or actin.

When Dbt^WT^ is expressed in fly head circadian cells in a *spag* RNAi background accumulation of activated Dronc is blocked (Fig 2A), suggesting that the effects of *spag* knock-down are mediated by reductions in Dbt. In addition, when Dbt is overexpressed in S2 cells it is able to block the accumulation of activated caspase associated with *spag* or *dbt* RNAi (Fig 2B). However, Spag overexpression was not able to block activated caspase accumulation brought on by *dbt* RNAi but did block activated caspase accumulation by *spag* RNAi, suggesting that Spag is upstream of Dbt and confirming the specificity of the dsRNAi knock-down for *spag* (Fig 2B).

### Lowered Spag and Dbt activity in circadian cells lead to accumulation of activated Dronc in the optic lobes via PDF signaling

**Fig 3:**
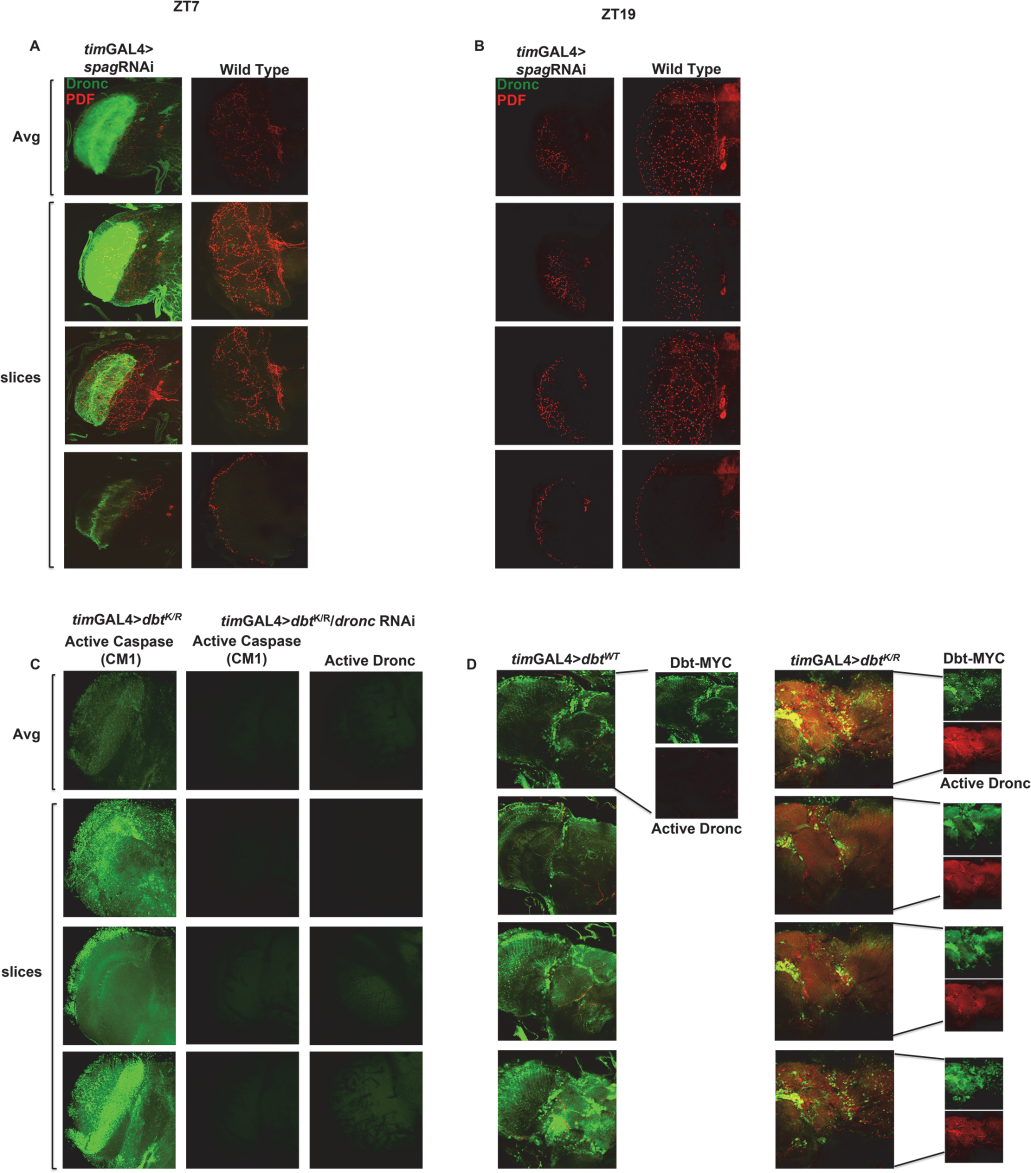
*Spag* knock-down or reduction in Dbt activity in clock cells induces activated caspase expression. (A) Knockdown of *spag* using the *tim*GAL4 driver induces accumulation of activated caspase. Whole brains were collected at ZT7 and active caspase (green) and PDF (red) were detected. The first image of each column shows the average intensity of the Z-stack image, followed by individual slices from the given Z-stack. Wild type controls showed no activated Dronc. (B) Whole brains were collected at ZT19 and PDF (red) but no active caspase (green) were detected in either genotype. (C) The caspase signal detected in fly brains is Dronc specific. Active caspase detected using anti-CM1 or anti-active Dronc at ZT7 in Dbt^K/R^ or Dbt^K/R^ and *Dronc* RNAi fly brains. (D) Caspase activation is detected in clock cells and non-clock cells. Whole brains were collected at ZT7 and imaged for Dbt-MYC (green) and active caspase (red), which was detected only in *tim*-GAL4>UAS-*dbt* ^*K/R*^ brains in both cells expressing Dbt-MYC and surrounding cells.

**Fig 4:**
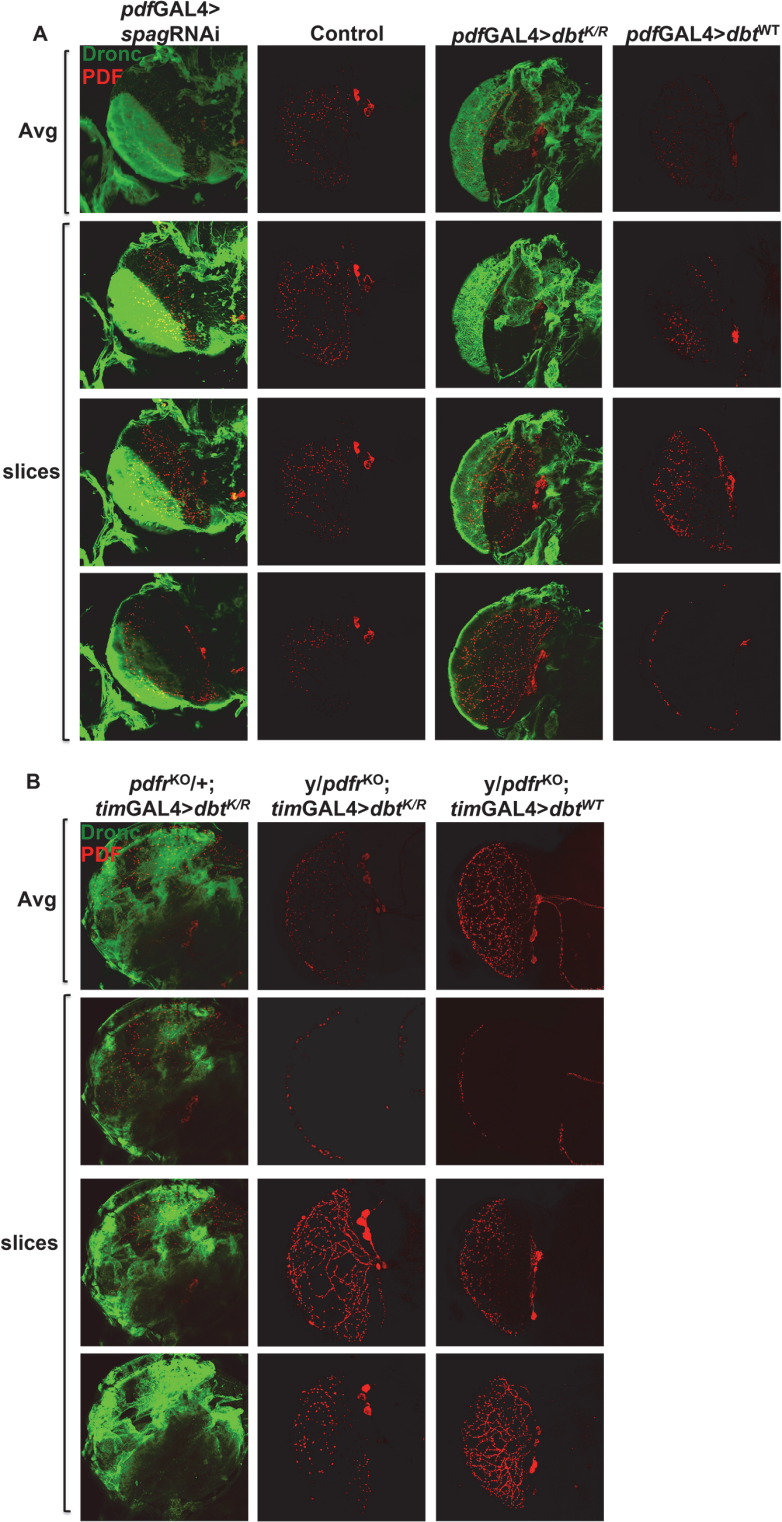
Pdf signaling from circadian neurons is required for accumulation of activated caspase in optic lobes. (A) Whole brains were collected at ZT7 from *pdf*GAL4>UAS-*spag*RNAi (or-*dbt* ^*K/R*^, -*dbt* ^*WT*^) flies and wild type control (*pdf*Tm3; progeny inheriting a balancer chromosome rather than the UAS responder) and processed for detection of PDF (red) and active caspase (green). Knock-down of *spag* or reduction of Dbt activity both produced activated caspase expression in the optic lobes in a region innervated by the PDF^+^ axons. (B) Whole brains were collected at ZT7 from *tim*GAL4>UAS-*dbt* ^*K/R*^ (or *dbt* ^*WT*^) flies and processed for detection of PDF (red) and active caspase (green). Flies that were heterozygous for the *pdf* receptor deletion (*pdfr* ^*KO*^/+) expressed activated Dronc with Dbt^K/R^, while those that were hemizgous for the mutation (*pdfr* ^*KO*^ males) did not. The first image of each column is the average intensity of the Z-stack image, followed by individual slices from the Z-stack. See S5 Fig for ZT19 analysis, which showed no expression of activated caspase under any condition.

Fly brains from *tim*GAL4>UAS-*spag* RNAi flies collected at ZT7 showed elevated levels of active caspase not found in control brains, but not at ZT19 (Fig 3A and 3B). This was also observed using the *pdf*GAL4 driver, which is expressed in the PDF-secreting brain neurons that drive circadian rhythms of locomotor activity in constant darkness, and with expression of the dominant negative Dbt^K/R^ with both drivers (Figs 3D and 4A for ZT7 and S5A for ZT19). *tim*-GAL4 clock cells expressing Dbt^K/R^ expressed both activated caspase and Dbt-MYC signal, but activated caspase was expressed in other cells as well (Fig 3D). Taken together, these results suggest both autonomous and non-autonomous effects of the transgene expression.

The elevated activated Dronc was particularly prominent in the optic lobe where the PDF^+^ axons terminate, but it was found in other brain-associated tissues as well. In addition, *Pdf* receptor mutant brains lacked this caspase activation that was observed at ZT7 (Figs 4B for ZT7 and S5B for ZT19). Moreover, most of the activated caspase was detected in tissue surrounding the PDF^+^ axons in the optic lobes (S5C Fig). Taken together with the generation of activated caspase in large areas of the brain in *pdf*GAL4>UAS-*spag*RNAi (or—*dbt* ^*K/R*^) flies, the results demonstrate that signaling by PDF is required for this broad activation pattern, and in fact it is even needed in an autocrine manner for expression in the PDF^+^ cells that also express PDF receptor [30], because no activation is seen in these cells in the absence of the PDF receptor (Fig 4B). The CM1 antibody, a marker for Caspase-9 like Dronc activity, was also used to detect Dronc activity, and showed a similar pattern as the anti-Dronc antibody used (Fig 3C). In addition, RNAi to Dronc eliminated most of the signal, confirming that the caspase signal we detect is indeed from Dronc (Fig 3C). Since all Dronc signal is eliminated with expression of *dronc* RNAi with the timGAL4 driver, the results suggest that broader Dronc activation requires Dronc activation in TIM^+^ cells, which then signal a corresponding increase in the surrounding tissue via PDF signaling.

### Tau is a substrate of Dronc when Dbt or Spag are knocked down

**Fig 5:**
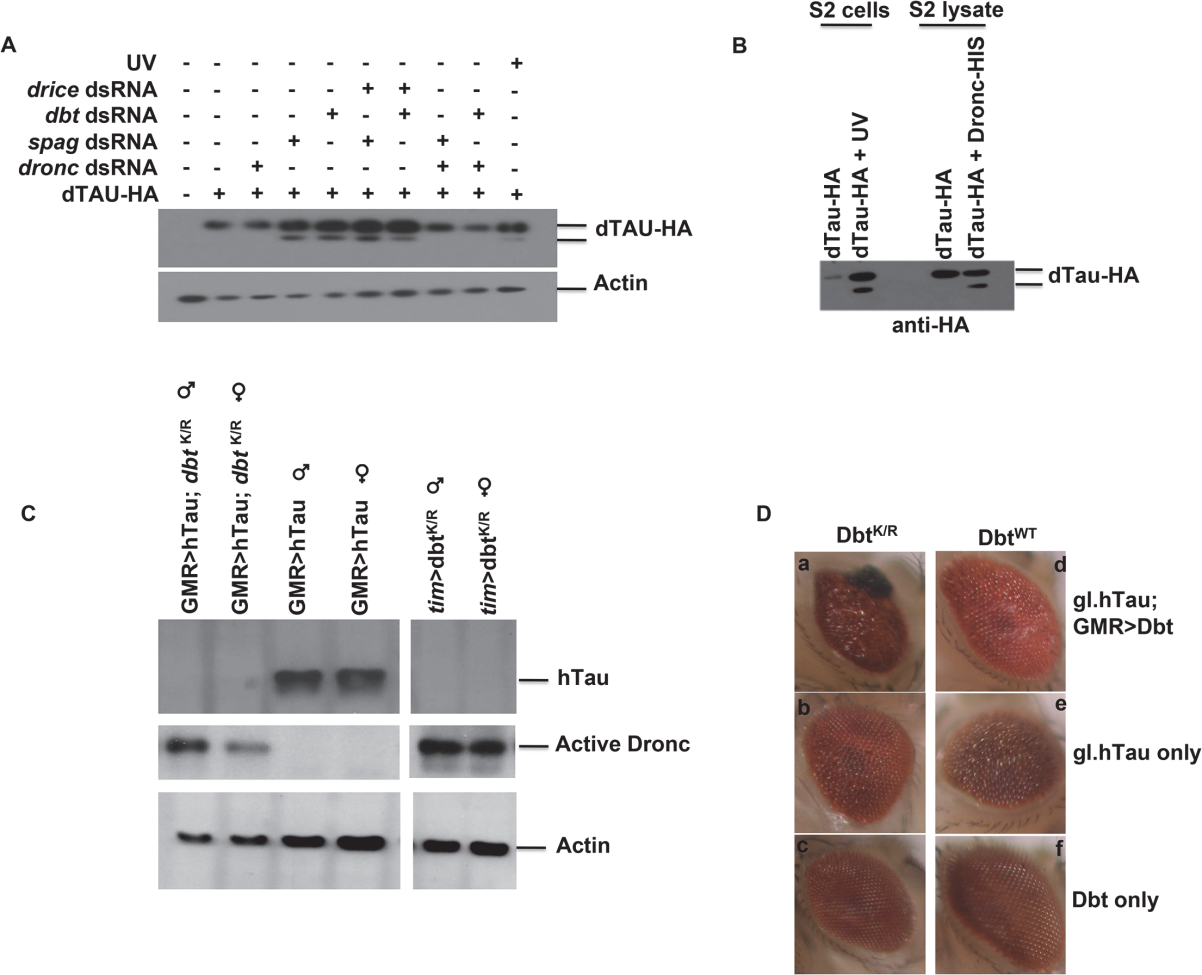
Spag or Dbt reduction leads to Dronc-dependent Tau cleavage and neurodegeneration in eyes overexpressing hTau. (A) S2 cells were transfected with Drosophila HA-tagged Tau +/- RNAi to *spag*, *dbt* alone or with either *dronc* or *drice* RNAi. Samples were then immunoblotted for dTau using anti-HA antibody. “UV” cells were treated with UV light to induce caspase activation, and this also produced cleavage of Tau. *dronc* RNAi inhibited Tau cleavage, while *drice* RNAi did not. (B) Drosophila HA-tagged *tau* was transfected in S2 cells and either UV treated to induce caspase activation or harvested and incubated with active recombinant Dronc. Samples were then immunoblotted for dTau using anti-HA. (C) Male and female flies were collected at ZT7 and immunoblotted for hTau, active Dronc and actin. Adult flies expressing Dbt^K/R^ in the eye with the indicated drivers in the presence or absence of hTau have activated Dronc, while expression of hTau alone did not produce activated Dronc. Flies expressing both hTau and Dbt^K/R^ show reduced Tau expression. (D) Adult flies expressing both Dbt^K/R^ and hTau produce an enhanced disrupted eye phenotype. Adult eyes of various genotypes were imaged and representative examples are shown. (a) gl.h*tau*; GMRGAL4>UAS-*dbt* ^K/R^ (b) GMRGAL4 >/+; gl.h*tau* (c) GMRGAL4>UAS-*dbt* ^K/R^ (d) gl.h*tau*; GMRGAL4>UAS-*dbt* ^WT^ (e) GMRGAL4 >/+; gl.*htau* (f) GMRGAL4>UAS-*dbt* ^WT^. See also S1 Table for quantification of eye size differences.

Since *spag* was initially found in a screen involving neurodegeneration and caspases have been implicated in the cleavage of key proteins associated with diseases such as AD, we examined whether Tau was a substrate for Dronc. First, we expressed HA-tagged Drosophila Tau (dTau) in S2 cells and either treated the cells with UV irradiation to induce widespread caspase activation or used RNAi to knock down either Spag or Dbt. When either Spag or Dbt was knocked down cleavage of dTau-HA was detected (Fig 5A). To confirm that Dronc was the main caspase involved in this cleavage we used RNAi and targeted either Dronc or Drice, the other main caspase involved in cell death. When Dronc was silenced along with Spag or Dbt, cleavage was inhibited, but when we targeted Drice the cleavage was still detected (Fig 5A).

To further confirm that Dronc was targeting dTau we incubated the dTau lysate with active recombinant Dronc. Samples from cells that were UV-irradiated showed a cleavage product for dTau. In addition, when active Dronc was used we also detected the same cleavage product (Fig 5B), which was not detected with lysates of S2 cells not treated with UV or active Dronc. This suggests that dTau is indeed a substrate for Dronc.

### Dbt^K/R^ expressed with hTau leads to activation of Dronc, cleavage of Tau and an enhanced disrupted eye phenotype

Expression of human Tau (hTau) in fly eyes produces neurodegeneration and has been used as a fly model for tauopathies [16,17]. Therefore, we expressed Dbt^WT^ or Dbt^K/R^ along with hTau in the fly eye using the eye specific GMR driver to determine if Dbt might enhance eye neurodegeneration. When Dbt^K/R^ was expressed activated Dronc was detected, both with and without hTau expression, while expression of hTau alone was not sufficient to activate Dronc (Fig 5C). Expression of DBT^K/R^ and hTau with GMR-GAL4 led to significant reductions in Tau levels at ZT7 (Fig 5C). Moreover, when Dbt^K/R^ was expressed along with hTau there was significantly increased disruption in the eye (Fig 5D and S1 Table). The enhanced disruption of the eye was manifested by the appearance of melanized patches in the eye (Fig 5D) and a decreased average surface area of the eye (S1 Table). Expression of Dbt^WT^ did not lead to enhancement of the eye phenotype (Fig 5D and S1 Table).

### Cell death-independent expression of activated Dronc in young flies and circadian effects on the cell death pathway in older flies

**Fig 6:**
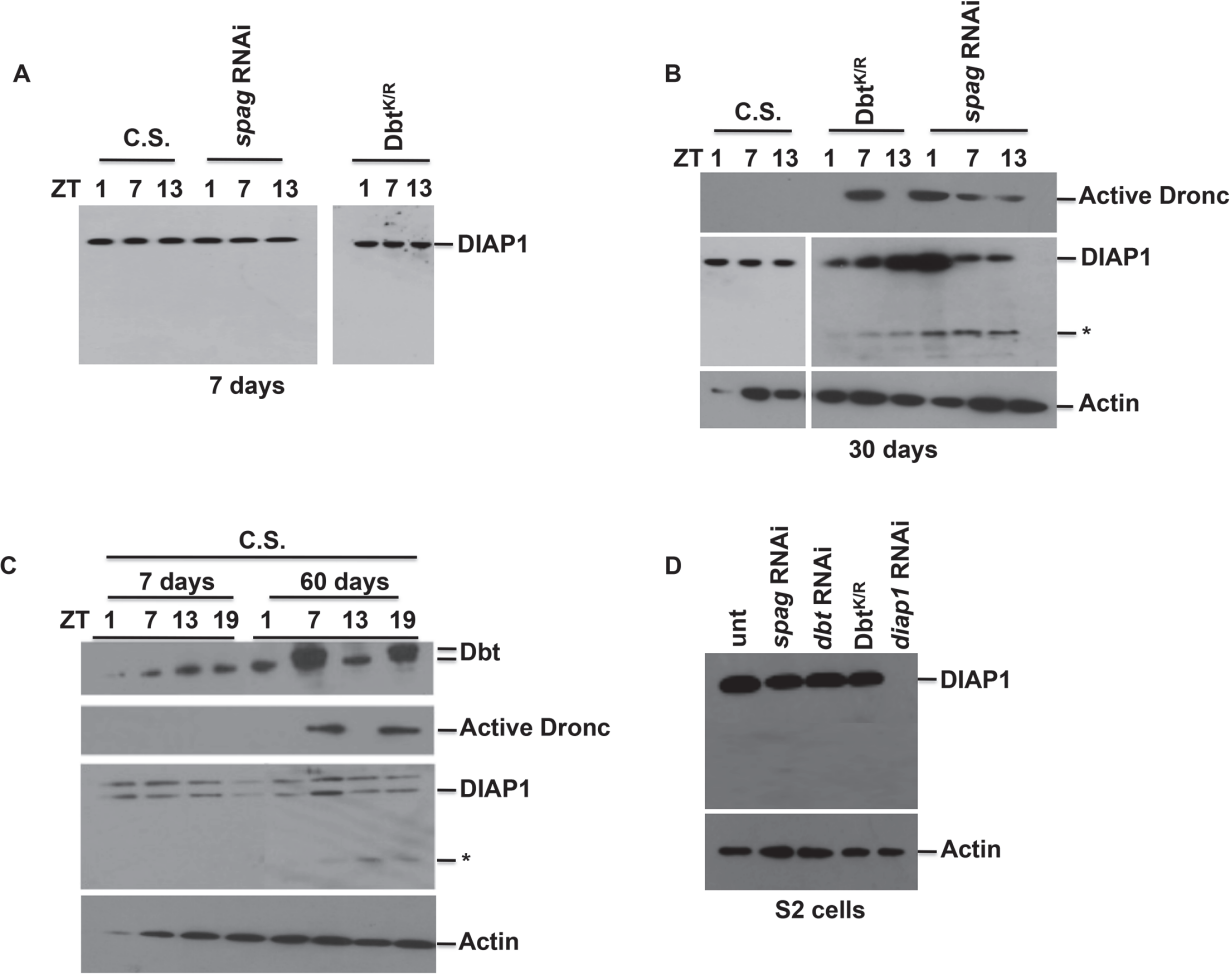
Apoptosis is not detected in young flies but is in older *spag* RNAi and wild type flies. (A) Fly heads were harvested from 7-day old or (B) 30-day old wild type flies Canton S flies (C.S.) or flies expressing the indicated transgene with the *tim*GAL4 driver at various times indicated and immunoblotted for Diap1, active Dronc or actin. Only 30-day old flies with reduced *spag* or *dbt* activity produced cleaved Diap1 (indicated by *), and *spag* RNAi flies produced active Dronc at all times at 30 days. (C) Fly heads from C.S. flies were collected at 7 and 60 days and immunoblotted for Dbt, active Dronc, Diap1 and actin. 60 day old C.S. flies showed Dbt mobility shift, active Dronc expression and Diap1 reduction at ZT7 and 19. Note that there were no *spag* RNAi flies still alive at this time. (D) S2 cells treated with *spag*, *dbt*, *diap1* dsRNA or stably expressing Dbt^K/R^ were immunoblotted for Diap1, demonstrating reduced Diap1 only with dsRNAi for *diap1*.

To determine whether Dronc activation led to classical apoptosis we looked at another marker of apoptosis. Diap1 is an antiapoptotic protein that regulates cell death in flies by binding to and inhibiting the activity of Dronc. When cell death occurs Diap1 is targeted for degradation/cleavage, thereby freeing Dronc [31]. We examined Diap1 levels in fly heads and observed no difference between young wild type and *tim*GAL4>UAS-*spag* RNAi or *dbt* ^*K/R*^ flies in which Dronc activation occurs (Fig 6A). This lack of effect on Diap1 levels was also observed in S2 cells treated with *spag* or *dbt* RNAi or expressing Dbt^K/R^ (Fig 6D). However, when flies aged a Diap1 cleavage product was detected in the *spag* RNAi and *dbt* ^*K/R*^ flies (Fig 6B) and activated caspase expression was also detected. Interestingly, aged *spag* RNAi flies showed activated caspase expression at all time points examined (Fig 6B). To determine if this pathway occurred naturally in aging flies we collected wild type Canton S (C.S.) fly heads at 60 days. Aged flies showed a mobility shift of Dbt at ZT 7 and 19 that was similar to what we observed in *spag* RNAi flies (Fig 6C). In addition, increased active caspase expression was detected at ZT7 and 19 for the aged flies (Fig 6C) and Diap1 cleavage was detected from ZT7 to 19 for the aged W.T. flies (Fig 6C). These results demonstrate that wild type flies exhibit the same circadian-dependent activation of apoptotic pathways that are produced in *spag* RNAi, *dbt* ^*K/R*^ and *Clk* ^*Jrk*^ flies at younger ages, and suggest that reductions in activity of these circadian genes accelerate an age-dependent pathway that leads to activation of the apoptotic pathway.

### *spag* RNAi flies have reduced climbing and lifespan

**Fig 7:**
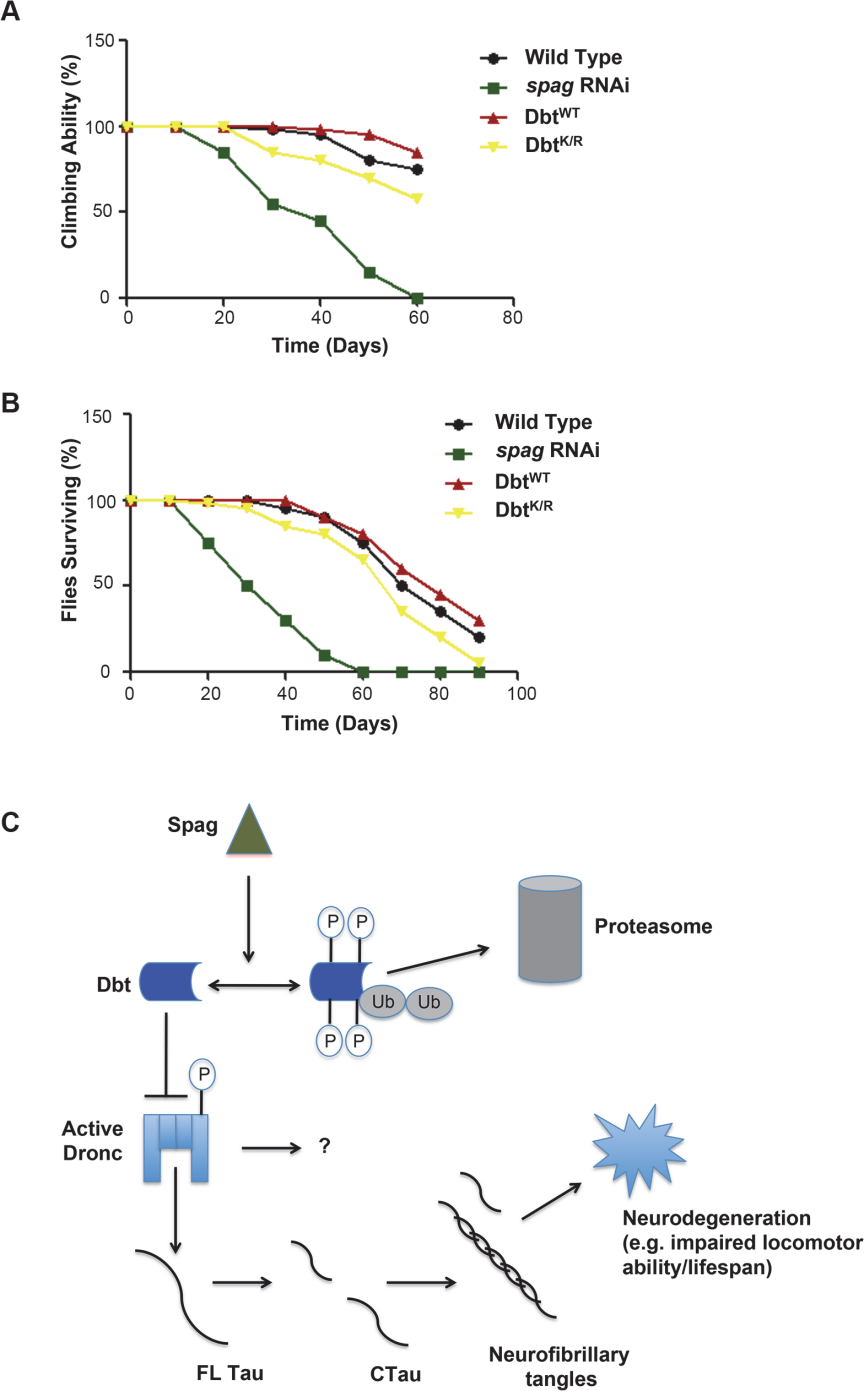
*spag* RNAi flies have a shorter lifespan and reduced climbing ability. (A) Climbing ability score. Quantification of the climbing performance of *spag* RNAi,-Dbt^WT^,-Dbt^K/R^ and control wild type flies as a function of age. Flies of each genotype were scored in three replicate groups of 10 for each genotype, and all showed the same trends. Genotypes: UAS-*dcr*; timGAL4>/UAS-*spag RNAi* (31253), *tim*GAL4>*dbt* ^WT^,-*dbt* ^K/R^, Canton S wild type flies. A Kruskal-Wallis nonparametric ANOVA indicated a significant effect of genotype on the time at which flies lost their climbing ability (H[3, N = 120] = 61, p<0.0001); the multiple comparisons p values indicated that the *spag* RNAi genotype differed significantly from all the others (p<0.0001), while the other three did not differ significantly. (B) Survival curves for the same genotypes as in (A). Flies were maintained at 25°C, transferred every 10 days in three replicate batches for each genotype, and scored for survival. All three replicates showed the same trend. A Kruskal-Wallis nonparametric ANOVA indicated a significant effect of genotype on lifespan (H3[N = 400] = 197, p<0.001). The multiple comparisons p values indicated that the *spag* RNAi genotype differed significantly from the other three while the *dbt* ^K/R^ genotype differed only from the *spag* RNAi and the *dbt* ^WT^ (p<0.002); no other comparisons differed significantly. (C) Model of Spag and Dbt regulation of caspases. Dbt regulates caspases by phosphorylation, which inhibits caspase activity in normal conditions. When *spag* is removed Dbt is targeted for phosphorylation and degradation by the proteasome, freeing the caspase and allowing for cleavage of Tau and possibly other substrates (“?”) and production of tauopathy

Since *spag* regulates a pathway that leads to the activation of caspases by reduction of Dbt, and this then leads to the cleavage of Tau by these activated caspases and ultimately to expression of apoptotic markers, we wanted to determine if there were behavioral and lifespan manifestations. The circadian locomotor assays initially suggested that there were effects as flies age. Locomotor behavior is preserved in young flies. However, as transgenic flies with reduction of *spag* by RNAi age, they tend to die during the locomotor assay (Table 1). The loss of the climbing response has been used to monitor age-related changes in Drosophila. Normal Drosophila show a strong negative geotactic response. When tapped to the bottom of a vial they rapidly climb to the top of the vial, and most flies remain there. Flies with knockdown of *spag* initially climb as well as control flies. However, over time they decline in performance more rapidly than controls (Fig 7A). The progressive, accelerated decline in climbing ability in *spag* RNAi flies demonstrates a functional deficit produced by knockdown of *spag* in clock cells. In addition, *spag* RNAi flies had a reduced lifespan compared to control flies (Fig 7B). By contrast, expression of Dbt^K/R^ in circadian cells did not produce accelerated death and loss of climbing ability in comparison with wild type Canton S flies, indicating that the effects of Dbt activity reduction are not as severe as those of *spag* reduction (See figure legend for discussion of statistics).

## Discussion

We have identified a new player (*spag*) that links circadian signaling, cell death and tauopathies together. Orthologs of Spag in yeast and humans function as co-chaperones of Hsp90 to regulate its activity and recruit client proteins for Hsp90. They are part of multiprotein complexes that contribute to biogenesis of cellular machineries like RNA polymerase, ribonucleoproteins and Phosphatidyl-Inositol 3-kinase-related kinases [32,33]. Recently, Drosophila Spag has been shown to associate with Hsp90 and Hsp70 to likewise contribute to assembly of several of these factors [34]. The human ortholog of Spag (RPAP3) is a binding partner for a WD40 repeat protein that is involved in apoptosis. RPAP3 contains TPR domains and regulates apoptosis induced by several stimuli [35,36].

When we knock down *spag* in circadian cells using RNAi we observed reduction in Dbt levels and an increase in the activated caspase Dronc in fly heads. Interestingly, this mostly occurred during the day (ZT7) or after extended light treatment at night, increased as flies age and did not occur in constant darkness. Accumulation of activated Dronc was also observed with expression of the kinase dead form of Dbt (Dbt^K/R^) in circadian cells. This suggests that some factor present or active during the light might regulate the Spag-Dbt pathway and confer a transient sensitivity to caspase activation after extended light treatment.

One feature that is common to various neurodegenerative diseases is the acceleration of the age-related disruption of the daily cycle of sleep and wake. Our work suggests that the most immediate way for the clock to influence neurodegeneration is by circadian gene-dependent control over the expression of pro-neurodegenerative factors. While these factors are not expressed in young flies with normal circadian clocks, the mutant clocks that we have produced in flies resemble those produced in aging wild type flies, in which Dbt modification, activated caspase expression and cleaved Diap1 are detected at ZT7 and ZT19. Prior work has shown the circadian function is blunted along with reduced healthspan in aging flies [37–39]. Circadian dysfunction would enhance their susceptibility to light-dependent neurodegeneration. In a previous report, the *period* gene was mutated in flies with *sniffer* gene mutations causing neurodegeneration. The flies with both mutations displayed faster neurodegeneration and had shorter lifespans compared to flies with single mutations. This suggests that disrupted circadian rhythms can accelerate the process of neurodegeneration [40].

What is the nature of this mechanism? Previous work linked Spag to Huntington’s disease and Hsp90 and a possible role in aggregation [25]. In addition, caspases have been shown to be involved in the cleavage of Tau, a protein associated with AD, and this caspase-mediated cleavage of Tau is associated with its aggregation. This led us to examine whether Tau was cleaved by the caspase Dronc in the Spag-Dbt pathway. HA-dTau was cleaved in S2 cells when we knocked down either Dbt or Spag, and this cleavage was prevented when we knocked down Dronc, but not the effector caspase Drice. This result is consistent with cleavage of Tau by Dronc in response to lowered Dbt and Spag activity.

We examined whether long-term reductions of Spag and Dbt activity in circadian cells have any detrimental effects on the fly. While younger flies with reduced Spag or Dbt activity did not exhibit expression of cell death marker Diap1, older flies with chronic reductions in Spag or Dbt activity exhibited elevated levels of Diap1 cleavage—a marker of the apoptotic pathway. These results suggest that chronic reductions in Spag or Dbt activity eventually produce deleterious effects.

Flies with reduced *spag* levels had more cleaved Diap1 compared to *dbt* ^K/R^ flies, higher levels of active Dronc and more rapid decline of climbing proficiency and lifespan. This puzzled us since both lines activate Dronc and lead to dTau cleavage, so we expected identical phenotypes. One possibility is that since Spag has been shown to interact with Hsp90, Spag might regulate Hsp90 and control the level of aggregation that occurs. In such a model, if Spag is eliminated and can no longer regulate Hsp90, Hsp90 might no longer interact with dTau and therefore no longer function to reduce dTau aggregates. The defect in Hsp90 function is not predicted for *dbt* ^K/R^ flies, which retain Spag. In addition, Hsp90 is a known regulator of cell death and has been shown to inhibit apoptosis [41]. Removal of Spag might lead to dysregulation of Hsp90, preventing it from regulating components of the cell death pathway and causing a higher level of activated caspase than observed with Dbt inhibition alone.

Is this pathway evolutionarily conserved? To address this we used the fly eye and expressed human Tau along with Dbt^WT^ or Dbt^K/R^. When hTau and Dbt^K/R^ were coexpressed together an enhanced disrupted eye phenotype was produced together with Donc activation and Tau cleavage, and the phenotype is less severe when hTau is coexpressed with Dbt^WT^. Since loss of Dbt kinase activity leads to Dronc activation, Dbt may be inhibiting Dronc by direct phosphorylation of Dronc, or alternatively there may be another intermediate target of Dbt. Prior work in mammals has demonstrated a link between reduced circadian clock function and neurodegeneration, as well as a link between CKIδ/ɛ and apoptosis [42–45]. Reduced clock function has been produced by alterations to circadian transcriptional regulators, with increased neurodegeneration produced in response to reactive oxygen species and induction of apoptosis. CKI regulation of cell death and cell cycle arrest has been linked to effects on the mitotic spindle, p53 and cell surface receptors involved in cell death. It is likely that circadian clocks and CKI affect apoptosis and neurodegeneration at multiple steps in addition to the ones outlined in this manuscript. However, the direct effects of the clock components on Dbt levels and the consequent expression of an activated initiator caspase suggest that these events may be upstream and global mediators of circadian effects on apoptosis and neurodegeneration.

It has been shown that these cell death components that are normally involved in destruction can also play critical roles in nonapoptotic events such as dendrite pruning, which occurs during development to create proper neural circuits. In Drosophila, caspase activity is detected locally in the degenerating dendrites and mutation of Dronc preserves most of the dendrite morphology [46]. In this instance caspases are not activated in the context of apoptosis, but in cell survival processes.

A possible role for the Spag-Dbt-Dronc pathway in dendritic or axonal pruning/remodeling is intriguing in light of the existing literature on the role of the circadian clock and light in these processes[47–50]. There is circadian remodeling of lateral neuron (PDF^+^) axon branching patterns as well as the size and synapses of several noncircadian neurons in the optic lobes, which are extensively innervated by the PDF^+^ axons. These circadian changes require a functional circadian clock, are enhanced by light, and in the case of some optic lobe changes require signals from the lateral neurons. Intriguingly accumulation of activated Dronc in the optic lobe at ZT7 in *tim*GAL4>UAS-*spag* RNAi (or UAS-*dbt* ^*K/R*^) flies also required light and was produced by PDF signaling from the lateral neurons; therefore, this activation may in fact be due to hyperactivation of the same pathways that trigger normal circadian neuronal remodeling in the optic lobes. In wild type flies, some of the optic lobe neurons exhibit largest axon size in the morning and the evening, suggesting that caspases (presumably below the level of detection) might contribute to pruning during the middle of the day, at times when activated caspases are detected here. It is not certain whether expression of activated Dronc in the optic lobe cells not expressing the *spag* RNAi (or *dbt* ^*K/R*^) also involves reductions in Dbt activity in those cells or is produced by a different pathway in response to PDF signaling, but it is likely that Dbt reductions in these cells also occur, as the reductions in Dbt detected in immunoblots of total head extracts can be quite complete. We would argue that PDF signaling is important for global DBT reductions, casapse activation and Tau cleavage, and that these are produced cell autonomously in S2 cells by *spag* RNAi or DBT^K/R^ expression. Reductions in *spag* may also trigger the non-cell autonomous reductions in Dbt levels and caspase activation, or these may be triggered by a *spag*-independent mechanism in response to *spag* decreases in the PDF^+^ cells. Caspase involvement in synapse degeneration has previously been suggested to contribute to AD pathologies [51].

We have identified a new mechanism of Tau cleavage and AD. We propose a model in which Spag regulates Dbt levels by regulating Dbt ubiquitination and/or phosphorylation, and when removed leads to the targeted degradation of Dbt. This removal of Dbt removes the inhibition on the caspase Dronc, leading to accumulation of its activated form and targeted cleavage of Tau (Fig 7C). This is the first identification of a mechanism activating caspases in the context of AD and other tauopathies and sheds new light on the underlying mechanism that regulates the disease state and its connection to the circadian clock. Recently, we identified another TPR-containing protein (Bride of Dbt, or Bdbt) that interacts with Dbt to enhance Dbt activity and regulate its phosphorylation state [29]. It will be interesting to determine if this protein is part of any Spag-Dbt complexes that might regulate AD and apoptosis. Furthermore, it will be important to establish the genetic, environmental or aging processes that could interact with the mechanism to activate it during normal aging to produce AD or potentially other outcomes that negatively impact health and lifespan.

## Materials and Methods

### Fly stocks

Drosophila RNAi stocks for spaghetti (CG13570) were obtained from the Bloomington Drosophila Stock Center (stock number: 31253, targeting nucleotides 778–1200 of the transcript) and the Vienna Drosophila RNAi Center (VDRC stock numbers: 23896, targeting nucleotides 886–1233 of the transcript; 103353KK, targeting nucleotides 780–1200). In addition, the *dronc* RNAi line 23033 was obtained from the VDRC. The expression of hTau was under the control of the *glass* (gl) promoter provided by George Jackson [17] for Fig 5D and S1 Table, or alternatively a UAS-hTau fly line from Mel Feany [16] was used to express human Tau with the GMR-GAL4 driver on the X chromosome (Bloomington stock center). The UAS-*dbt-myc* lines and the *tim*GAL4 and UAS-*dcr*; *tim*GAL4 driver lines have been described previously [27,28]. The *pdf*GAL4 driver line (stock number 6899) and the *Pdf* receptor (CG13758) null mutant line (*Pdfr* ^*5304*^; line 33068) were obtained from the Bloomington Drosophila Stock Center. *Clk* ^*Jrk*^ [52], *per* ^*o*^, *per* ^*S*^ and *per* ^*L*^ mutations [53] were used alone or together with the *dbt* ^*K/R*^ constructs as described in the text.

### Locomotor activity analysis

UAS-*spag RNAi* lines were crossed with UAS-*dcr2*; *tim*Gal4 lines. Progeny were then continuously reared at 23°C in a 12 hr:12 hr LD cycle for one more week after collection of adults (or longer where indicated) to insure complete RNAi knock-down effects, and loaded onto monitors (Trikinetics, Waltham, MA) for behavioral monitoring in constant darkness (DD) for at least 5 days. Actogram activity records and periodogram analysis to determine periods were employed as previously described with ClockLab [27].

### Immunoblot analysis

Fly heads were collected at ZT 1, 7, 13, and 19 or S2 cells at the times indicated in 1X SDS loading buffer, homogenized and stored at -80°C until analyzed. Samples were subjected to SDS-PAGE, transferred onto nitrocellulose and probed with appropriate antibodies: anti-DbtC (1:2000) [27], anti-activated Dronc (1:100) [26], anti-Actin (1:1000) (Developmental Studies Hybridoma Bank), anti-Diap1 (1:100) [54], anti-Tau (Developmental Studies Hybridoma Bank) and anti-HA (1:500) (Covance PRB-101P), and signals were detected with the appropriate secondary antibodies and the ECL detection procedure. Anti-activated Dronc was purified by protein affinity purification. Antisera were applied to a protein G bead column bound with full length inactive Dronc and the flow-through was collected. This was followed by incubation of the flow-through with protein G beads bound with active Dronc. The beads were eluted with 0.1M glycine, pH 2.7 and used.

### Adult brain immunofluorescence

Brains were dissected from flies at ZT7 and ZT19, fixed, permeablized and incubated with anti-Dronc (1:100), anti-CM1 (Cell Signaling #9661), anti-PDF (1:5000) (PDF C7, Developmental Studies Hybridoma Bank) or anti-Myc (1:1000) (Santa Cruz sc-789-G) antibody overnight at 4°C. The following day samples were incubated with fluorescently-labeled secondary antibodies (anti-rabbit IgG Alexa fluor 488 or anti-mouse IgG Alexa fluor 568, 1:1000). The brains were examined using an Olympus Fluoview confocal, and Z stacks were obtained.

### Drosophila Tau cleavage assay

Drosophila S2 cells were transiently transfected with dTau-HA and 24h later cells were treated with dsRNA corresponding to *dronc*, *dbt*, *spag* or *drIce* and harvested 48h later. For UV treatment, cells were harvested 24h later. Samples were analyzed by immunoblot using anti-HA antibody.

For treatment with active Dronc-His, S2 cells were harvested 24h after dTau-HA transfection, resuspended in Buffer A (25mM Tris·Cl pH 8.0, 50mM NaCl, 10mM DTT) and lysed by 4x freeze-thaw. After lysis, cells were spun down at 13000rpm and the supernatant was collected and incubated with active Dronc-His [54] for 4h at 30°C. The reaction was stopped by the addition of 5X SDS loading buffer and analyzed for dTau-HA by immunoblot analysis. As a positive control, extracts were analyzed from S2 cells expressing dTau-HA and treated with UV light.

### Quantification of eye size in flies expressing hTau and/or Dbt-MYC

Fly eye size was measured from photomicrogaphs using the ImageJ program (open source program, NIH). A circumference was drawn around the eye and the area was obtained from the measure command. The values (number of pixels) were then tabulated and averaged and statistics were performed using the Statistica (Statsoft OK) software.

### RT-PCR analysis

Fly heads (50–100) or S2 cells were collected and frozen in liquid nitrogen. Total RNA was isolated using the Trizol Reagent (Invitrogen), and cDNA synthesis was performed using the Taqman Reverse Transcription Kit (Life Technologies). PCR was performed using gene specific primers for *spag* and actin for 20 cycles, and the products were analyzed on an agarose gel.

### Spag and Dbt rescue or overexpression experiments

S2 cells stably expressing Spag or Dbt were untreated or treated with dsRNA corresponding to *spag* or *dbt*. Forty eight hours after dsRNA addition cells were harvested and analyzed for active Dronc, Dbt or actin. Plasmids used for this experiment were the following: pMT-*dbt-myc*, pMT-*dbt* ^*K/R*^ *-myc*, pAC-*spag-ha*.

### Primers used to generate dsRNA and the procedure for treatment of S2 cells with dsRNAi

*dbt* (nt 321–674):

forward 5’-TAATACGACTCACTATAGGGGCGCGTGGGTAACAAATATC-3’,

reverse 5’-TAATACGACTCACTATAGGGTGTATGTAATCGATGCGGGA-3’,

*dronc* (nt 939–1454):

forward 5’-TAATACGACTCACTATAGGGATGGTGGGGATAGTGCCATA-3’, reverse 5’-TAATACGACTCACTATAGGGTGTCAGGCCACTTCTCCTCT-3’,

*drIce* (nt 653–968):

forward 5’- TAATACGACTCACTATAGGGACTGCCGCTACAAGGACATT-3’,

reverse 5’- TAATACGACTCACTATAGGGGCGTGCACTGGAATCTTGTA-3’,

*diap1*(nt 491–1019):

forward 5’-TAATACGACTCACTATAGGGCCGGCATGTACTTCACACAC-3’, reverse 5’-TAATACGACTCACTATAGGGTTCTGTTTCAGGTTCCTCGG-3’,

*spag* set 1 (nt 735–1242):

forward 5’-TAATACGACTCACTATAGGGCAAAAGTGGGCCAAACTTTAC-3’

reverse 5’TAATACGACTCACTATAGGGTTCTGGGCTGCGTTCTAT-3’,

*spag* set 2 (nt22-227):

forward 5’-TAATACGACTCACTATAGGGGGAGCGCTAGCAACAGAAAT-3’

reverse 5’-TAATACGACTCACTATAGGGGCGACTTCTGGAGCTCTTTC-3’,

PCR fragments were produced from Drosophila genomic DNA with these primer sets. dsRNAi was produced and transfected into S2 cells by the procedures of the Perrimon lab (http://www.flyrnai.org/DRSC-HOME.html), using the T7 promoters encoded by the primers. The cells were harvested at the times indicated in the figure legends. In one experiment, the proteasome inhibitor MG132 was added to a concentration of 50–100 μM, and in another MYC-tagged Dbt was overexpressed.

### Generation of *spag* expression clones

A cDNA clone for CG13570 (RE03224) was obtained from the Drosophila Genomics Resource Center (DGRC, Bloomington, IN). We used the DGRC Gateway collection in order to clone *spag* into vectors allowing expression in S2 cells. A full-length *spag* ORF was generated by PCR (Phusion, cat# F-553S, NEB) and cloned into pENTR/SD/D-TOPO (Cat# K240020, Life Technologies) by TOPO-mediated cloning. This clone was used to generate plasmids containing an HA tag. The clonase enzymatic mixture (Cat# 11791–019) was purchased from Life Technologies. *spag* was cloned into the pGateway vector pAHW (Cat#1095) to generate an N-terminal 3xHA-tag plasmid. The plasmid were driven by an Act5c promoter allowing for constitutive expression in S2 cells.

### Generation of dTau-HA plasmid

For the expression of Drosophila Tau (dTau) in S2 cells, the pFLC1-TAU cDNA (Drosophila Genomics Resource Center, Clone # RE16764) was cloned into the pAc S2 cell expression vector. The N-terminal HA tag was added using the following primers:

Tau N-TER Forward, 5’-GCGCGAATTCGCGACCCTATGGCGTACCCGTACGACGTGCCGGACTACGCGATGGCGGATGTCCTGGAGAAAAGCTCACTG 3’,

Tau N-TER DRA Reverse, 5’—GGCGCATGTCCGACTTGTACC 3’

The DNA was digested with DraIII and EcoRI and swapped for the original fragment in the pAc-Tau.

### Climbing and lifespan assays

Flies were maintained at 24°C on a 12 hr:12hr light/dark cycle. For aging studies, virgin male flies were isolated and maintained in 20 per vial and transferred to a fresh vial every 10 days. The number of dead flies was recorded.

For the climbing assay, flies were sorted into groups of ten per vial and tested. A climbing apparatus was prepared for each group, with two empty polystyrene vials vertically joined by tape facing each other. For the lower vial, we measured a vertical distance of 8 cm above the bottom surface and marked each vial by drawing a circle around the entire circumference of the vial. We transferred a group of ten flies into the lower vial and allowed the flies to acclimatize to the new setting for 1 minute before conducting the assay. Flies were gently tapped down to the bottom of the vial and the number of flies that climbed above the 8 cm mark by 10 seconds after the tap was measured[55]. This was repeated with the same group ten times, allowing for a 1 minute rest period between each trial. The average number of flies per group that passed the 8-cm mark as a percentage of total flies was recorded.

## Supporting Information

S1 Fig *spag* is required in circadian cells to produce rhythmic behavior. Representative DD actogram records of progeny with the indicated genotypes are presented (left), along with periodogram analysis to determine the period of the rhythm (right). Knock-down of *spag* led to a longer period (B, D) or arhythmicity (C) when compared to control (A). Flies were assayed starting two weeks after collection of adults, except for panel D, for which the record started one week after collection.
(PDF)

S2 Fig Analysis of the parameters affecting Dbt post-translational modification and degradation triggered by *spag* RNAi. This analysis demonstrated that Dbt reduction is variable and accentuated by higher temperatures and light, with a post-translational modifications of Dbt observed with shorter periods of *spag* RNAi treatment or in younger flies. (A) The specificity of the purified anti-activated Dronc antibody (See Materials and Methods) for the active cleaved form of Dronc using recombinant full length and active cleaved Dronc. (B) S2 cells were treated with *spag* dsRNA, harvested at 1 and 2 hours after dsRNA addition and immunoblotted for Dbt. (C) *tim*GAL4>*spag* RNAi fly heads from young (3 days) and old (7 days) collections were immunoblotted for Dbt. (D) *tim*GAL4>*spag* RNAi flies were reared at 24 or 27°C, and fly heads were harvested at 7 days or 14 days after collection at the indicated times and immunoblotted for Dbt. (E) Constant darkness blocks Dbt reduction caused by knock-down of *spag* in fly heads. Flies expressing *spag* RNAi with the *tim*GAL4 driver were entrained in a light/dark cycle or kept in constant darkness, collected at the indicated days and times (ZT7 in LD or CT7 in DD) and examined for Dbt levels. (F) Flies were light pulsed for 7 hours at night (starting at ZT13) and Dronc activation was determined by immunoblot.
(PDF)

S3 Fig Confirmation of *spag* knock-down and effects in various lines. (A) RT-PCR analysis of *spag* transcript in various *spag* RNAi lines and in S2 cells. (B) Analysis of different *spag* RNAi lines for effects on Dbt and caspase activation. Fly heads from *tim*GAL4>UAS-*spag* RNAi lines with different RNAi constructs were isolated at the indicated times of day, and immunoblotted for Actin, Dbt and activated Dronc. Dbt was consistently reduced and Dronc was consistently activated at ZT7, while Dbt was variably reduced at ZT19 without detection of activated Dronc. The numbers above the lanes are the numbers for the relevant line from the Vienna Drosophila Resource Center (VDRC).
(PDF)

S4 Fig Time course analysis of Dronc activation in various mutant genotypes. Heads of adult wild type Canton S flies (C.S.) and of the indicated mutants were collected at the indicated times and immunoblotted for active Dronc and actin.
(PDF)

S5 Fig Brain confocal images collected at ZT19 showing no expression of activated caspase. (A) Whole brains from the indicated genotypes were collected at ZT19 and active caspase (green) and PDF (red) were detected. The top image for each column depicts the average intensity of the Z-stack image with each additional image representing an individual slice from the Z-stack. (B) Pdf receptor mutants at ZT19 expressing wild type or catalytically inactive Dbt (Dbt^K/R^) collected at ZT19. Whole brains were collected and active caspase (green; not detected at this time) and PDF (red) were assayed. The first image of each column depicts the average intensity of the Z-stack image and each image below that depicts individual slices from the Z-stack. (C) A single optical section magnified to show detection of activated caspase and PDF in the optic lobes of *tim*GAL4>UAS-*spag* RNAi flies at ZT7. Most of the activated caspase is detected in areas surrounding the PDF^+^ axons rather than in the axons themselves.
(PDF)

S1 Table Average Areas of Fly Eyes Expressing hTau and Dbt. (DOCX)
