# Supplementary figures and images for: Drosophila Spaghetti and Doubletime Link the Circadian Clock and Light to Caspases, Apoptosis and Tauopathy
Source: PLoS Genet. 2015 May 7;11(5):e1005171. doi: 10.1371/journal.pgen.1005171 (PMC4423883; doi:10.1371/journal.pgen.1005171)

**A****Wild Type**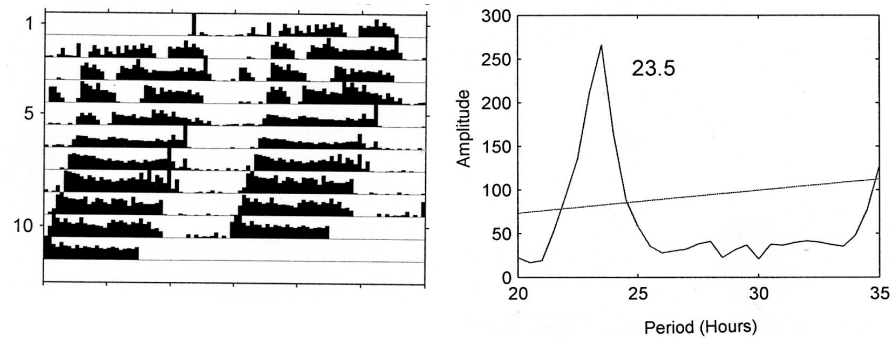**B****UAS-*dcr2*; *tim*GAL4>/23896RNAi**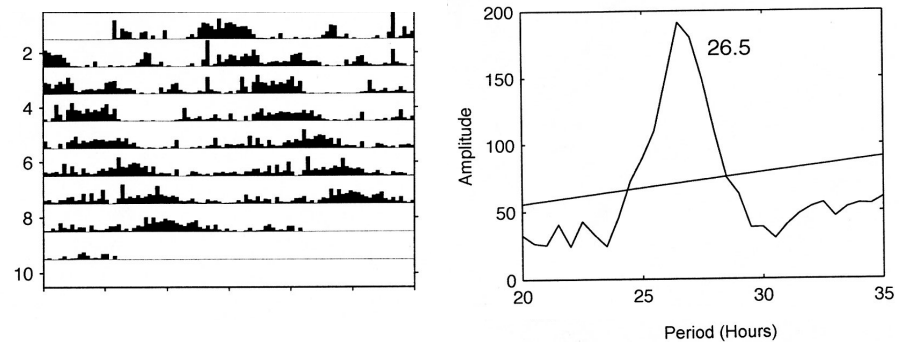**C****UAS-*dcr2*; *tim*GAL4>/31253RNAi**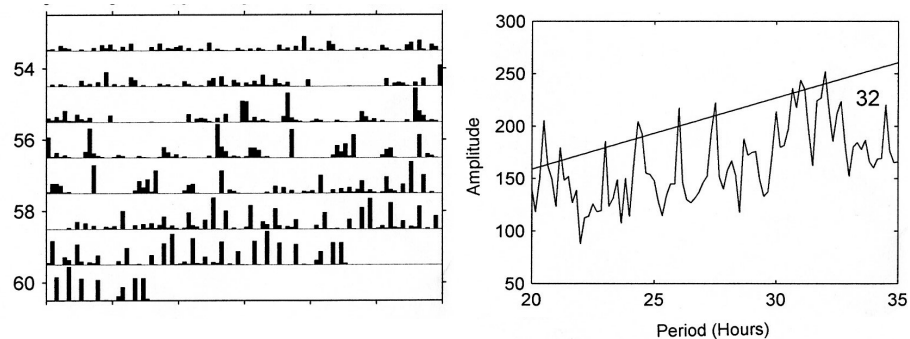**D****UAS-*dcr2*; *tim*GAL4>/103353kkRNAi**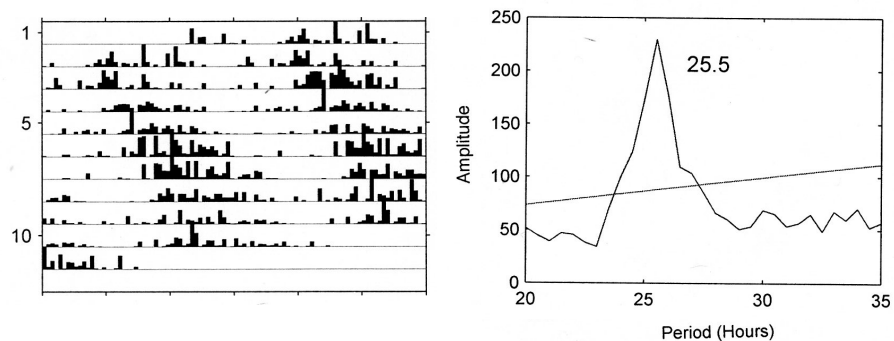**1 week**

Supplement: S1 Fig — Representative DD actogram records of progeny with the indicated genotypes are presented (left), along with periodogram analysis to determine the period of the rhythm (right). Knock-down of spag led to a longer period (B, D) or arhythmicity (C) when compared to control (A). Flies were assayed starting two weeks after collection of adults, except for panel D, for which the record started one week after collection. (PDF) [file pgen.1005171.s001.pdf]

**A**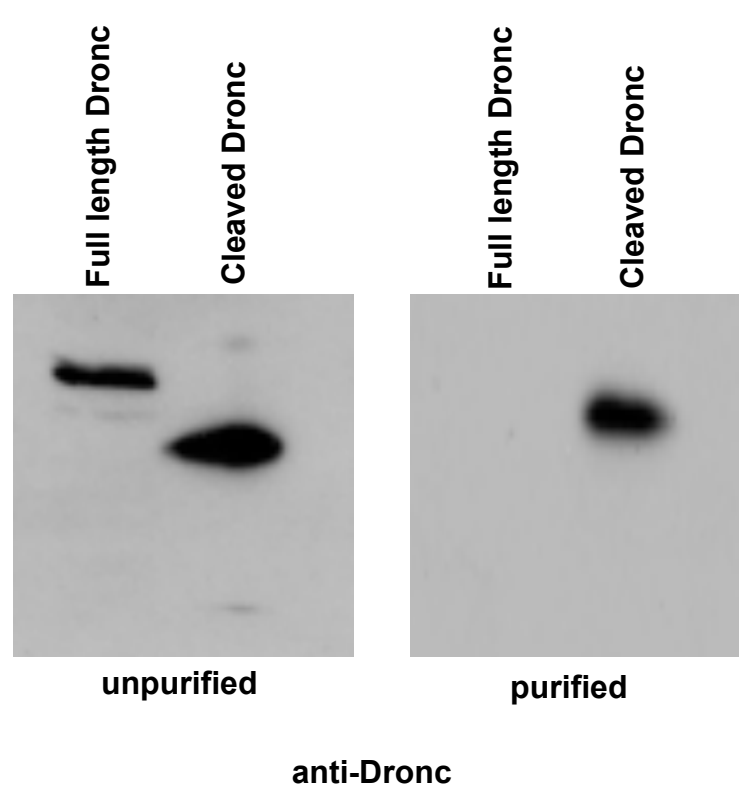**B**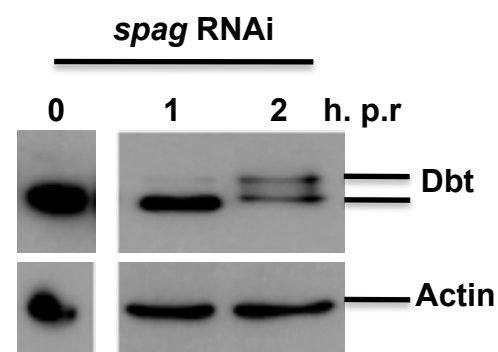**C**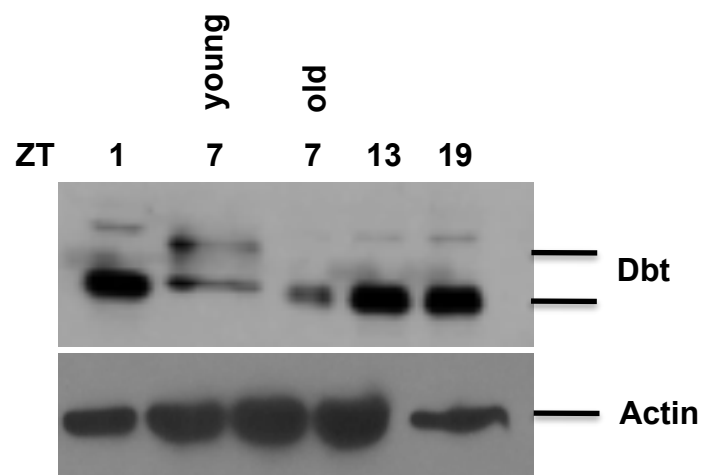**D**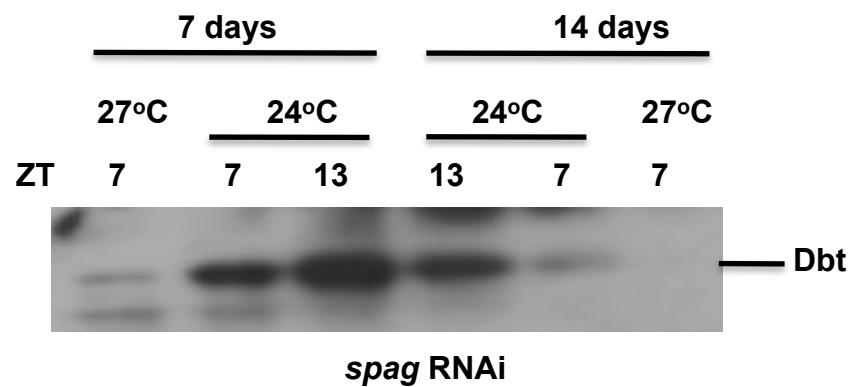**E**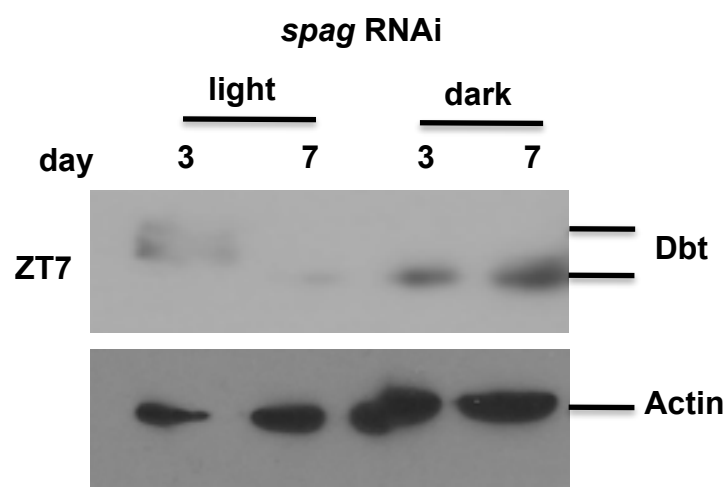**F**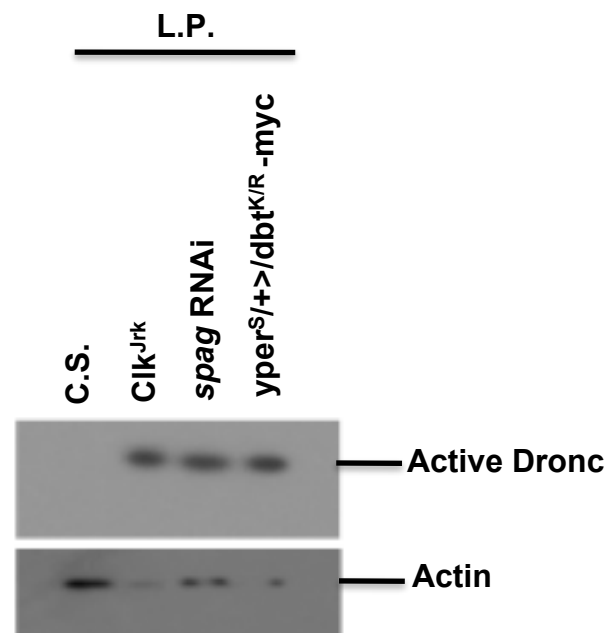

Supplement: S2 Fig — This analysis demonstrated that Dbt reduction is variable and accentuated by higher temperatures and light, with a post-translational modifications of Dbt observed with shorter periods of spag RNAi treatment or in younger flies. (A) The specificity of the purified anti-activated Dronc antibody (See Materials and Methods) for the active cleaved form of Dronc using recombinant full length and active cleaved Dronc. (B) S2 cells were treated with spag dsRNA, harvested at 1 and 2 hours after dsRNA addition and immunoblotted for Dbt. (C) timGAL4>spag RNAi fly heads from young (3 days) and old (7 days) collections were immunoblotted for Dbt. (D) timGAL4>spag RNAi flies were reared at 24 or 27°C, and fly heads were harvested at 7 days or 14 days after collection at the indicated times and immunoblotted for Dbt. (E) Constant darkness blocks Dbt reduction caused by knock-down of spag in fly heads. Flies expressing spag RNAi with the timGAL4 driver were entrained in a light/dark cycle or kept in constant darkness, collected at the indicated days and times (ZT7 in LD or CT7 in DD) and examined for Dbt levels. (F) Flies were light pulsed for 7 hours at night (starting at ZT13) and Dronc activation was determined by immunoblot. (PDF) [file pgen.1005171.s002.pdf]

**A**

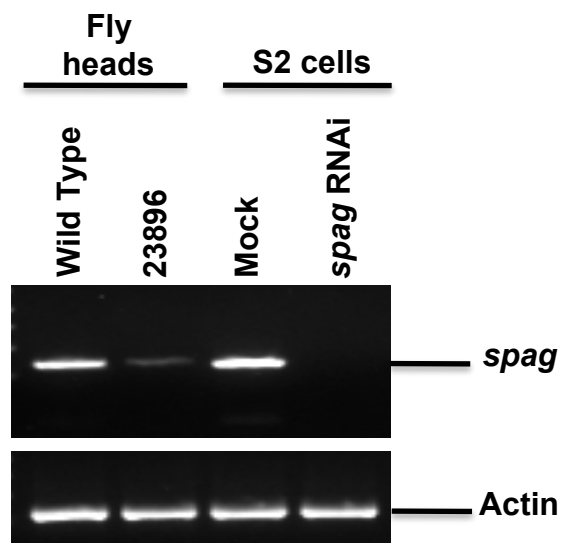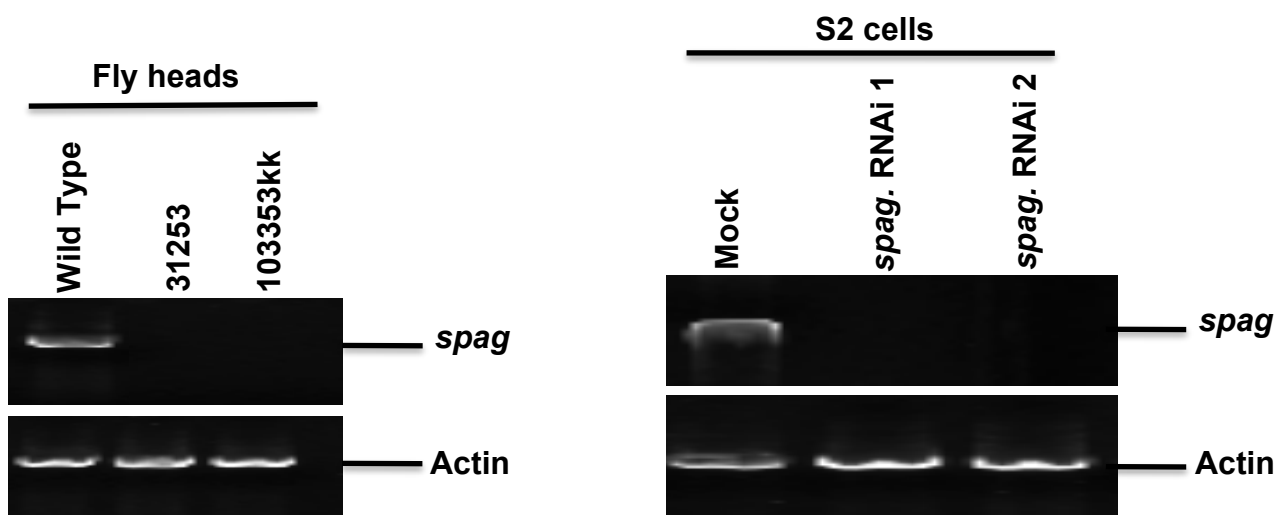

**B**

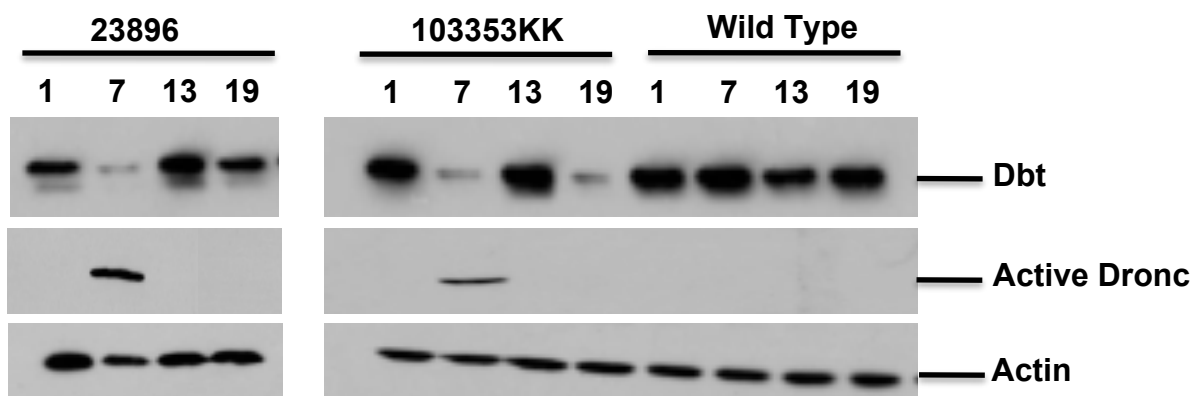

Supplement: S3 Fig — (A) RT-PCR analysis of spag transcript in various spag RNAi lines and in S2 cells. (B) Analysis of different spag RNAi lines for effects on Dbt and caspase activation. Fly heads from timGAL4>UAS-spag RNAi lines with different RNAi constructs were isolated at the indicated times of day, and immunoblotted for Actin, Dbt and activated Dronc. Dbt was consistently reduced and Dronc was consistently activated at ZT7, while Dbt was variably reduced at ZT19 without detection of activated Dronc. The numbers above the lanes are the numbers for the relevant line from the Vienna Drosophila Resource Center (VDRC). (PDF) [file pgen.1005171.s003.pdf]

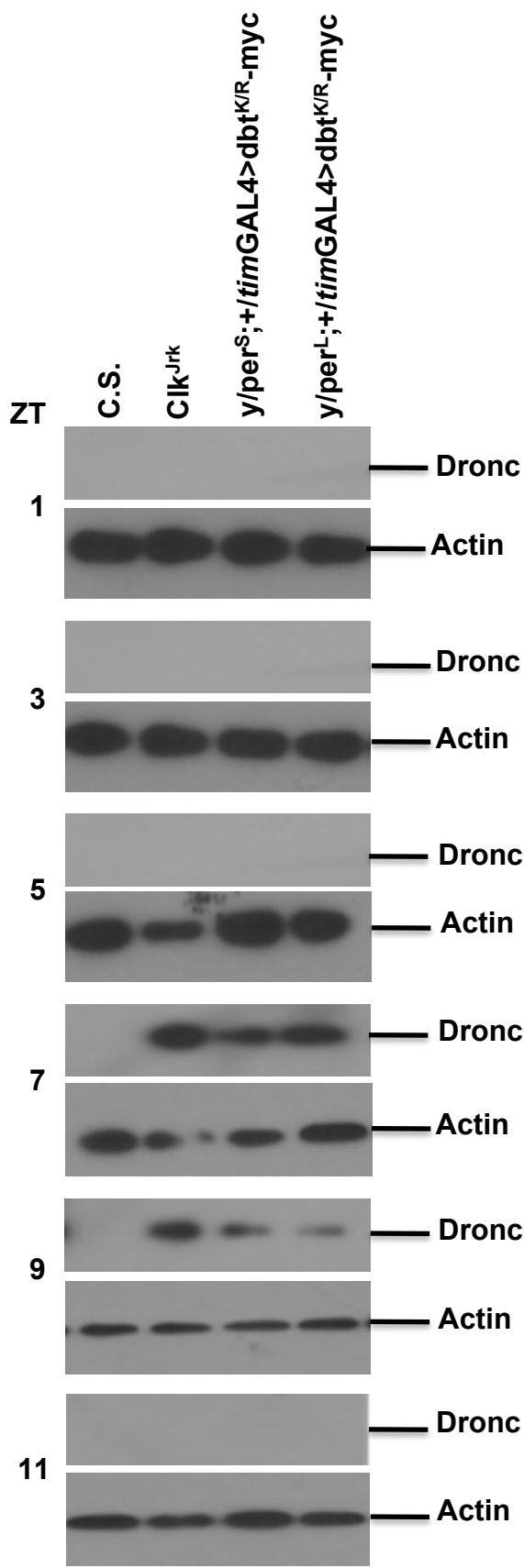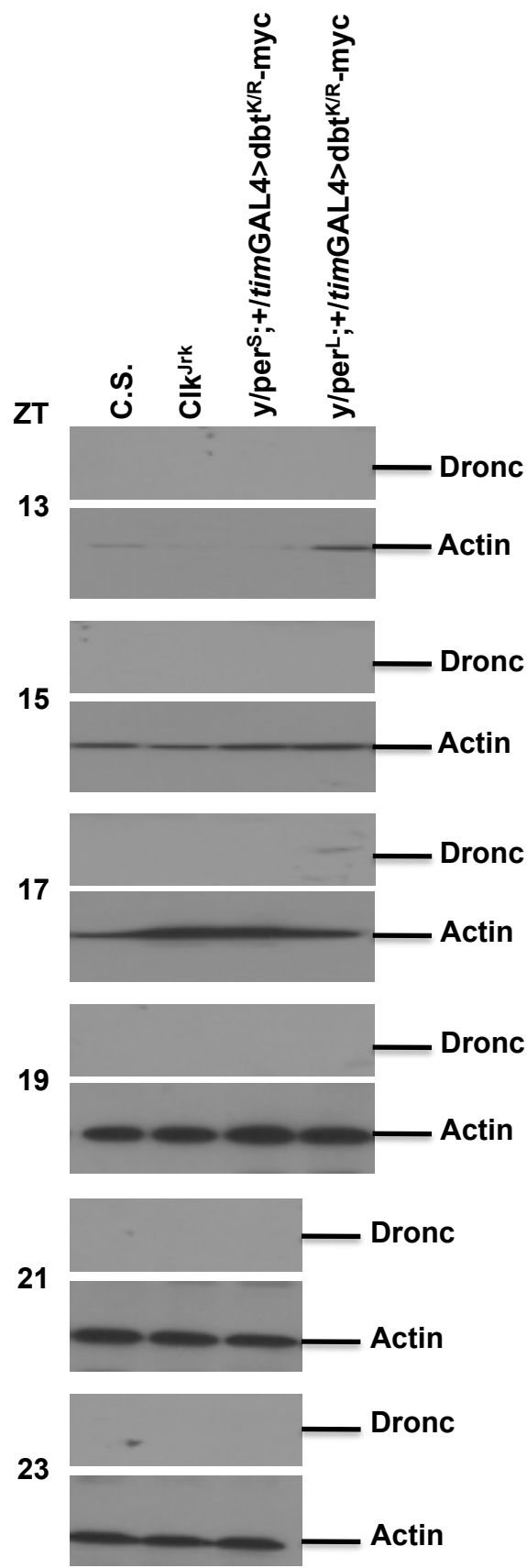

Supplement: S4 Fig — Heads of adult wild type Canton S flies (C.S.) and of the indicated mutants were collected at the indicated times and immunoblotted for active Dronc and actin. (PDF) [file pgen.1005171.s004.pdf]

A

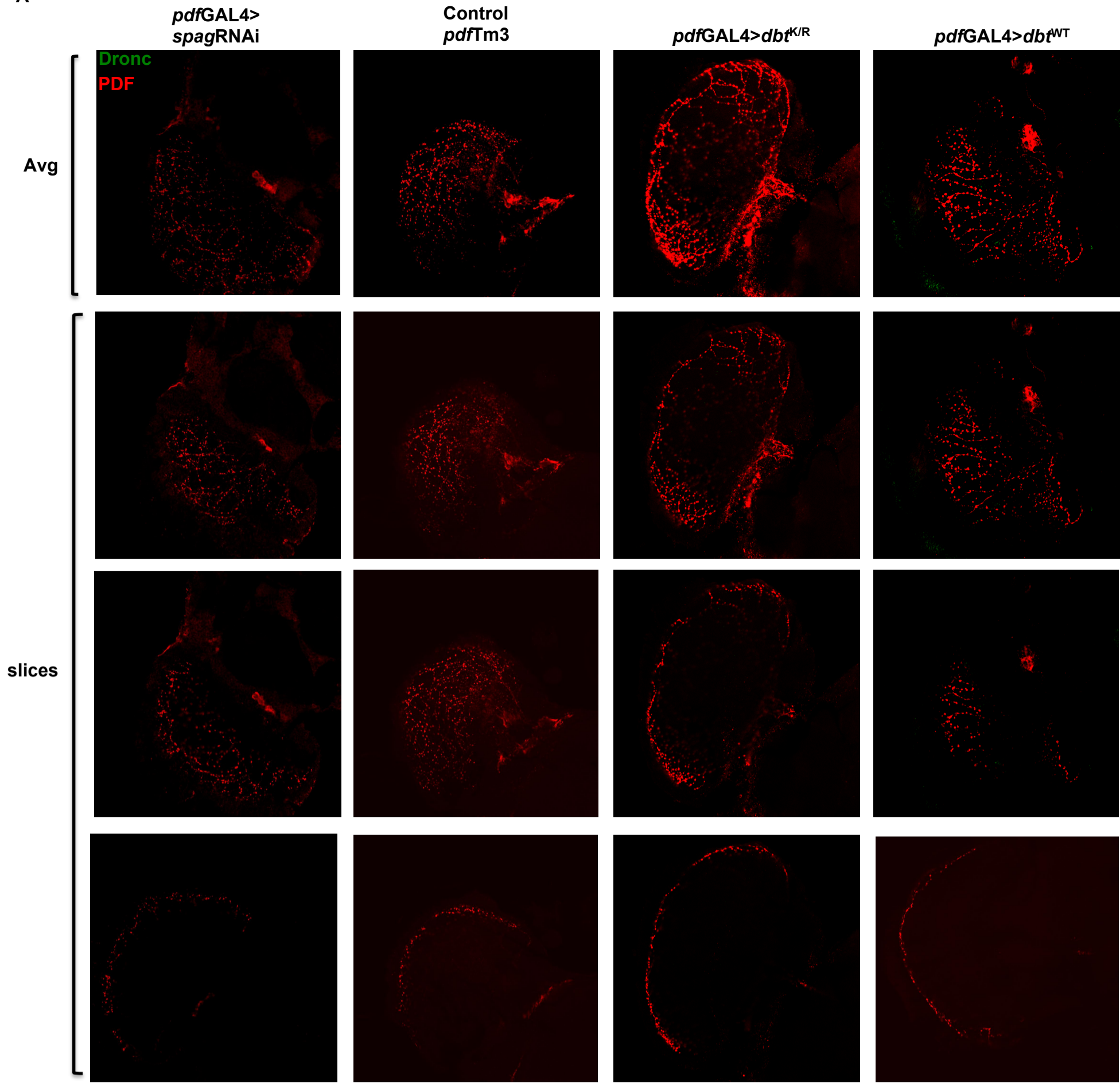

B

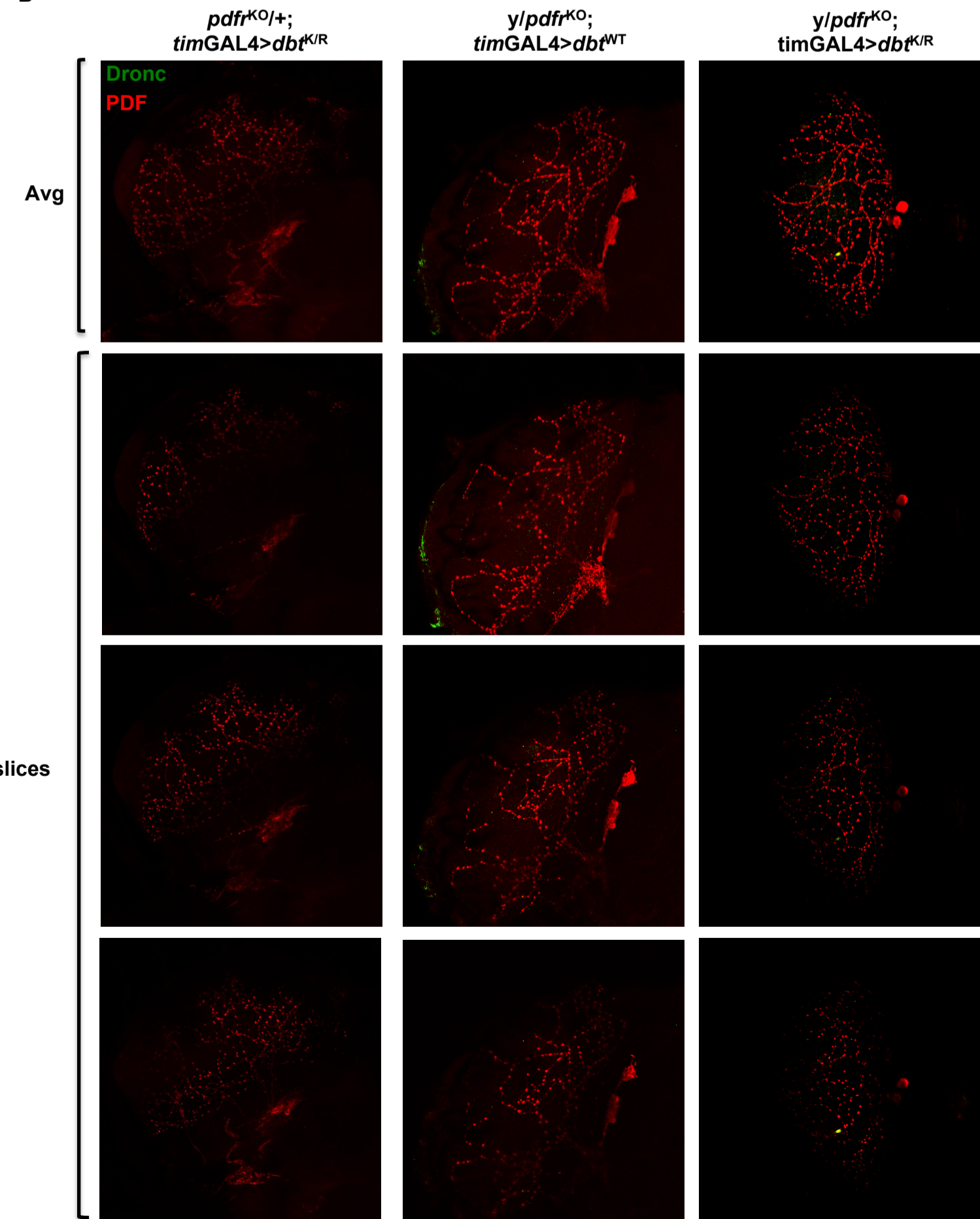

C

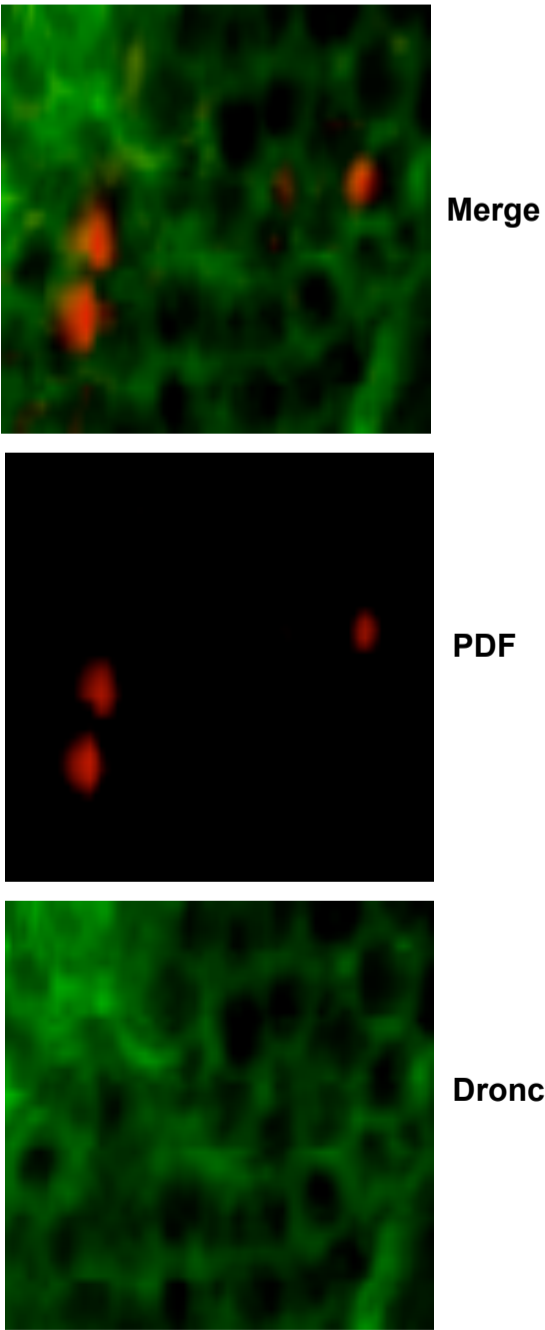

Supplement: S5 Fig — (A) Whole brains from the indicated genotypes were collected at ZT19 and active caspase (green) and PDF (red) were detected. The top image for each column depicts the average intensity of the Z-stack image with each additional image representing an individual slice from the Z-stack. (B) Pdf receptor mutants at ZT19 expressing wild type or catalytically inactive Dbt (DbtK/R) collected at ZT19. Whole brains were collected and active caspase (green; not detected at this time) and PDF (red) were assayed. The first image of each column depicts the average intensity of the Z-stack image and each image below that depicts individual slices from the Z-stack. (C) A single optical section magnified to show detection of activated caspase and PDF in the optic lobes of timGAL4>UAS-spag RNAi flies at ZT7. Most of the activated caspase is detected in areas surrounding the PDF+ axons rather than in the axons themselves. (PDF) [file pgen.1005171.s005.pdf]
