# Supplementary material for: Drosophila Spaghetti and Doubletime Link the Circadian Clock and Light to Caspases, Apoptosis and Tauopathy
Source: PLoS Genet. 2015 May 7;11(5):e1005171. doi: 10.1371/journal.pgen.1005171 (PMC4423883; doi:10.1371/journal.pgen.1005171)
Supplement: S1 Table — (DOCX) [file pgen.1005171.s006.docx]

________________________________________________________________________

**Table S1. Average areas of fly eyes expressing hTAU and DBT**

**________________________________________________________________________**

**Progeny No of Flies Average eye size SD**

**________________________________________________________________________**

*gl*-TAU; GMRGAL4 > DBT^K/R^ 22F1B* 30 76391 11411
*gl*-TAU; GMRGAL4 > CyO 22F1B 9 102731 13036
SM6TM6; DBT^K/R^ 22F1B 14 104978 15519

*gl*-TAU; GMRGAL4 > DBT^K/R^ 24F1B* 13 72599 12819
*gl*-TAU; GMRGAL4 > CyO 24F1B 10 98168 15019
SM6 TM6; DBT^K/R^ 24F1B 7 119726 19396

*gl*-TAU; GMRGAL4 > DBT^WT^ 45F2B 19 114567 18549
SM6 TM6 > DBT^WT^ 45F2B 13 132767 18926

*gl*-TAU; GMRGAL4 > DBT^WT^ 21M2B 11 125645 9005
*gl*-TAU; GMRGAL4 > CyO 21M2B 12 104656 16584
SM6 TM6; DBT^WT^ 21M2B 7 130090 23956

GMRGAL4 > DBT^K/R^ 22F1B 19 125870 18435
GMRGAL4 > CyO 22F1B 19 132047 11443

GMRGAL4 > DBT^K/R^ 24F1B 12 127360 16475

________________________________________________________________________

Fly eyes were outlined in Image J using the free hand tool and the area was calculated. Areas (numbers of pixels in the eyes) were calculated for the number of flies indicated on the table for each genotype and averaged for the data shown on table. ANOVA indicated a significant effect of genotype [F(13,181) = 27.2, P<0.001]. * Mean eye size of these flies significantly differed from that of gl-tau; GMRGAL4>UAS-DBT^WT^ and other controls by post-hoc Tukey.
